# Quantitative chemometric phenotyping of three-dimensional liver organoids by Raman spectral imaging

**DOI:** 10.1016/j.crmeth.2023.100440

**Published:** 2023-03-31

**Authors:** Vernon LaLone, Aleksandra Aizenshtadt, John Goertz, Frøydis Sved Skottvoll, Marco Barbero Mota, Junji You, Xiaoyu Zhao, Henriette Engen Berg, Justyna Stokowiec, Minzhi Yu, Anna Schwendeman, Hanne Scholz, Steven Ray Wilson, Stefan Krauss, Molly M. Stevens

**Affiliations:** 1 Department of Materials, Department of Bioengineering and Institute of Biomedical Engineering, Imperial College London, London SW7 2AZ, UK; 2 Hybrid Technology Hub-Centre of Excellence, Imperial College London, London SW7 2AZ, UK; 3 Hybrid Technology Hub-Centre of Excellence, Institute of Basic Medical Sciences, Faculty of Medicine, University of Oslo, P.O. Box 1112, Blindern, 0317 Oslo, Norway; 4 Department of Chemistry, University of Oslo, P.O. Box 1033, Blindern, 0315 Oslo, Norway; 5 Department of Pharmaceutical Sciences, University of Michigan, Ann Arbor, MI 48109, USA; 6 Biointerfaces Institute, University of Michigan, Ann Arbor, MI 48109, USA; 7 Department of Immunology and Transfusion Medicine, Oslo University Hospital, P.O. Box 4950, Nydalen, 0424 Oslo, Norway; 8 Department of Transplant Medicine, Oslo University Hospital, Oslo, Norway; 9 Institute for Surgical Research, Oslo University Hospital, Oslo, Norway

**Keywords:** drug metabolism, hepatotoxicity, liver organoids, organoid benchmarking, Raman spectral imaging, quantitative chemotyping, qRamanomics

## Abstract

Confocal Raman spectral imaging (RSI) enables high-content, label-free visualization of a wide range of molecules in biological specimens without sample preparation. However, reliable quantification of the deconvoluted spectra is needed. Here we develop an integrated bioanalytical methodology, qRamanomics, to qualify RSI as a tissue phantom calibrated tool for quantitative spatial chemotyping of major classes of biomolecules. Next, we apply qRamanomics to fixed 3D liver organoids generated from stem-cell-derived or primary hepatocytes to assess specimen variation and maturity. We then demonstrate the utility of qRamanomics for identifying biomolecular response signatures from a panel of liver-altering drugs, probing drug-induced compositional changes in 3D organoids followed by *in situ* monitoring of drug metabolism and accumulation. Quantitative chemometric phenotyping constitutes an important step in developing quantitative label-free interrogation of 3D biological specimens.

## Highlights

• qRamanomics allows calibrated, quantitative chemometric analysis of 3D biospecimen
• qRamanomics enables multi-spectral benchmarking of liver organoids
• qRamanomics reveals multi-spectral changes in drug-treated liver organoids
• qRamanomics detects drug metabolites in liver organoids *in situ*

## Motivation

Quantitative chemometric imaging tools for validating the composition, functional maturity, disease progression, and response to therapeutic interventions are of significant interest in the rapidly expanding organoid arena. Raman spectral imaging allows high-content, label-free detection of tell-tale biomolecules, but it requires reliable quantification of deconvoluted spectra to unfold its full potential.

## Graphical abstract

**Figure:**
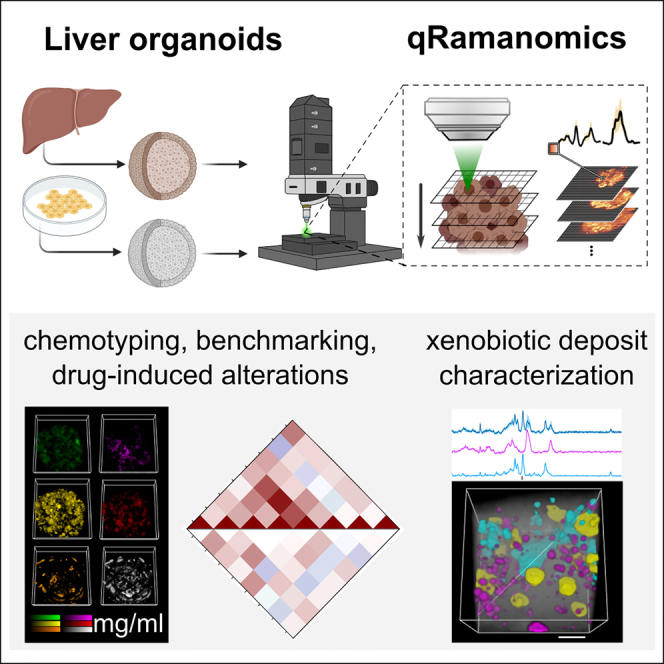

## Teaser

LaLone and Aizenshtadt et al. present qRamanomics, a bioanalytical platform for non-invasive quantitative chemometric phenotyping of 3D biological specimens by confocal Raman spectral imaging. The platform was applied for benchmarking of iPSC-derived liver organoids, for describing drug-induced compositional changes in liver organoids, and for *in situ* monitoring of drug metabolism and accumulation.

## Introduction

There is a significant need for reliable human organ representations (termed organoids) that provide bio-relevant model systems with foreseeable utility in disease modeling, drug discovery, and personalized drug testing. Induced pluripotent stem cell (iPSC) technology enables *in vitro* development of human organoids that show features of the organs they represent. However, organoids typically lack the structure and functional maturity of their human counterparts and show significant intra- and inter-batch variations. Hence, there is a need for advancing multi-factorial organoid characterization, including their response to therapeutic interventions, by applying high-content and high-resolution imaging tools.^1^

Conventional fluorescence-based confocal imaging and *in situ* hybridization are the methods of choice for visualizing localization and dynamics of biomolecules at cellular and subcellular levels, based on specific labels. However, while researchers have a broad selection of probes to mark specific proteins and nucleic acid sequences, other classes of biomolecules including carbohydrates, lipids, and metabolites are in general more difficult to visualize. Moreover, because of a so-called color barrier, only a limited number of targets can be investigated simultaneously in a specimen by most label-dependent techniques.^2^ In addition, *in situ* quantification of biomolecules is challenging and can only be done indirectly and in relative units (e.g., mean fluorescence intensity).

Confocal Raman spectral imaging (RSI), measuring predominantly the vibrational modes of molecules, can enable high-content label-free visualization of a wide range of molecules (including carbohydrates, lipids, proteins, nucleic acids, specific metabolites, drugs, and minerals) in biological specimens without sample preparation. While this phenomenon may be exploited in fixed and living tissues as the emitted spectral information is reported to remain consistent,^3^ recording times for Raman spectra make current studies in fixed tissue the standard.

Recently, RSI has been applied for assessing cell differentiation^4^^,^^5^^,^^6^ and for quantitative characterization of tissue-engineered and native cartilage and bone.^7^^,^^8^^,^^9^ Furthermore, the utility of RSI was demonstrated through direct measurements of drugs and drug metabolites, with subcellular resolution, in selected cell lines including cancer cells (BaF3/BCR-ABL1, SK-BR-3, NCI-H1975, Calu-3) and macrophages (raw 264.7).^10^^,^^11^^,^^12^^,^^13^ Despite this progress, the full potential of biospecimen analysis by RSI is hindered by demanding unmixing of signals derived from complex biological matrices. In addition, direct quantification of biomolecules by RSI is possible in principle, but so far has been challenging. As a significant step to address these challenges, quantitative volumetric Raman imaging (qVRI) was previously developed for analysis of individual mesenchymal stem cells in 3D biomaterials^14^ offering volumetric insight into the size of subcellular features without, however, providing information on local concentrations of biomolecules. Quantification at the single-cell and subcellular levels remains a topic of debate throughout the Raman community and is complicated by the absence of reliable calibration standards and standardized preprocessing algorithms.^14^^,^^15^^,^^16^

Building on the work of LaLone et al.^17^ and Kuzmin et al.,^18^ here we present the development of a robust calibrated bioanalytical platform for quantitative chemometric phenotyping by confocal RSI of biological specimens ranging from single cells to complex 3D organoids. The methodology, which we term quantitative Ramanomics (qRamanomics), allows direct structural and quantitative compositional characterization of biological specimens in absolute biochemical measurements with subcellular spatial resolution.^19^ We apply qRamanomics to iPSC-derived 3D hepatic organoids, benchmarked to primary human hepatocyte spheroids, with the goal of interrogating their state of maturation, response to drug challenges, and drug metabolism. Our work addresses a currently unmet analytical need and paves the way for further advances in supervised organoid development and tracking.

## Results

### Development of a platform for quantitative chemometric phenotyping of 3D biospecimens: qRamanomics

**Figure 1:**
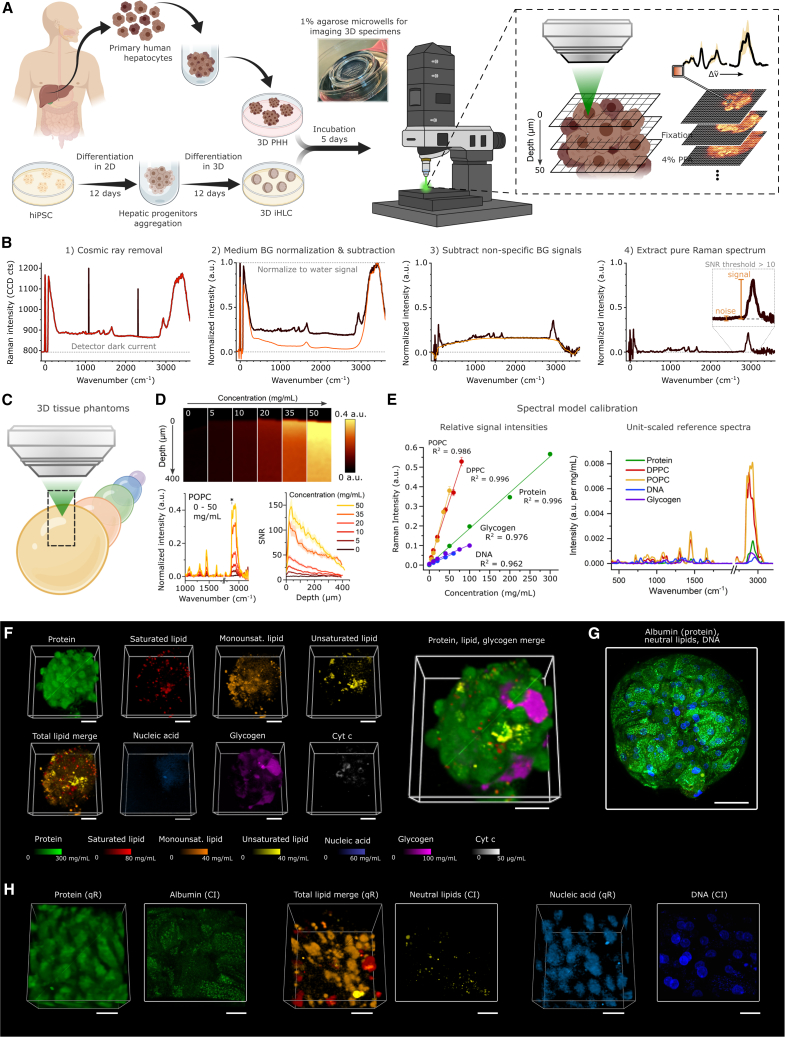
qRamanomics platform for chemometric phenotyping of 3D biospecimens enables quantitative assessment of distribution, abundance, and co-localization of biomolecules (A) Workflow for the formation and Raman analysis of 3D PHH spheroids and 3D iHLC organoids. (B) Spectral preprocessing algorithm employing the water signal as an internal standard for all measurements. (C) Scheme of Raman analysis of 3D biomolecular tissue phantoms of varying composition. (D) 3D tissue phantoms with range of POPC concentrations elucidate linear range of quantitation and allow for depth-dependant signal interference studies. (E) Signal intensities vary for each major class of biomolecules, and reference spectra are scaled accordingly to extract the unit-scaled (a.u. per 1 mg/mL) spectra for each. (F) High-content qRamanomics imaging of the whole 3D PHH spheroid. Scale bars, 50 μm. (G) Representative image of the whole organoid immunostaining for albumin with counterstaining of neutral lipids and DNA. Scale bar, 50 μm. (H) High-resolution qRamanomics imaging (qR, left) and confocal imaging (CI, right) of protein, lipids, and nucleic acids in the segment of the 3D iHLC organoid. Scale bars, 20 μm.

To develop a platform for quantitative chemometric phenotyping of hepatic organoids by confocal RSI (Figure 1A), we designed a 3D tissue phantom calibration technology. This calibration methodology enables direct simultaneous measurement of the absolute local concentrations of the most abundant biomolecular components and sequestered xenobiotics in organoids.

First, we identified a robust preprocessing algorithm to normalize and correct the Raman spectra for liquid water (Figure 1B). Employing the OH stretch band in the spectra of liquid water (3,400 cm^−1^),^20^ all analyte Raman signals were normalized relative to the aqueous matrix, thereby accounting and correcting for singularities of each 3D data voxel. Next, common biomolecular components of eukaryotic cells were formulated as single-component and multi-component tissue phantoms (Figures 1C and S1A) containing a range of concentrations of proteins (10–300 mg/mL), saturated (5–80 mg/mL) and monounsaturated (5–50 mg/mL) lipids, DNA (10–60 mg/mL), and glycogen (10–100 mg/mL) in PBS. Cytochrome *c* (Cyt *c*) was included in the quantitative model by dissolving Cyt *c* in PBS to elucidate the concentration-dependent intensity of the spectra (a.u. per μg/mL). The poor solubility of lipids in aqueous media was resolved by employing formulated saturated (1,2-dipalmitoyl-*sn*-glycero-3-phosphocholine [DPPC]) and monounsaturated (1-palmitoyl-2-oleoyl-*sn*-glycero-3-phosphocholine [POPC]) synthetic high-density lipoprotein nanodisks (sHDLs) of ∼10 nm particle diameter as previously described^16^ (Figure S1A). The nanoscale particle size distribution of DPPC and POPC sHDLs was comparable with that of albumin and substantially smaller than in conventional tissue phantom intra-lipid emulsions as revealed by dynamic light scattering (DLS). This allowed dissolution of lipids up to 80 mg/mL and facilitated miscibility with other biomolecules at varying concentrations in PBS.^21^

To assess the depth-dependent depletion of scattered Raman signals, 3D phantoms were subsequently analyzed in reflected analysis mode in the z dimension (exemplified by single-component tissue phantoms containing POPC in Figure 1D). Acquired data served as a quantitative calibration standard for Raman cytometry, elucidating sample homogeneity and signal interference profiles as a function of 3D imaging depth whereby the signal-to-noise ratio (SNR) was calculated as a function of depth across various concentrations and compositions of tissue phantoms. The calibration was repeated across concentration ranges for protein, DPPC, POPC, DNA, and glycogen, yielding linear relationships across relevant ranges of concentrations. To estimate normalized Raman intensity per mg/mL, unit-scaled reference spectra were generated from linear relationships of pre-processed spectra across bio-relevant concentration ranges (Figure 1E). The complete unit-scaled reference spectral model accurately identified and deconvoluted single-component tissue phantom data with minimal errors using linear combination modeling approaches. Bayesian model fitting enabled specificity and precision confidence assessments across measured concentration ranges (Figure S1B) in complex multi-component mixtures (Figure S1C). The two most important sources of potential error in this calibration system were the accuracy of actual concentrations in the calibration standards (i.e., “ground truth”) and misfitting errors associated with statistical unmixing of pre-processed Raman spectra. Regardless, the fundamental linear theory presented here establishes a robust framework for quantitative chemotyping analysis of 3D biospecimens.

Next, qRamanomics was applied to primary human hepatocyte spheroids (3D PHH) to establish a chemometric tool for label-free 3D characterization and as a benchmarking standard for iPSC-derived hepatocyte-like cell containing organoids (3D iHLC) (Figure S2A). 3D Raman hyperspectral image datasets were acquired via continuous scanning of laser voxels in a raster pattern across a series of z-stacked x-y image planes achieving optimal possible spatial resolution of ∼500 nm in the x-y plane and ∼1 μm in the z plane. In this setting, the depth of reliable data acquisition was limited to 50 μm in the z plane (corresponding to approximately 2–3 cell layers and hence able to penetrate 25% of an average 3D PHH) to ensure adequate SNR for accurate spectral unmixing and quantification. The methodology allowed reliable acquisition of up to 30,000 spectra per organoid specimen (Figure S1D) with subcellular spatial resolution (Figure S1E). All endmember reference spectra were unit-scaled according to the calibration model (Figure 1E) and used to translate the spectral data into local concentration measurements of proteins, lipids (saturated, monounsaturated, unsaturated), nucleic acids (combined DNA and RNA due to overlapping spectra), glycogen, and Cyt *c* in the organoids. The quantitative data were subsequently merged into a 3D representation showing the spatial distribution and relation of each component in 3D PHH (Figure 1F). The Raman data of chemometric deconvoluted 3D PHH could then be compared with labeled confocal images obtained from similar 3D PHH showing albumin, neutral lipids, or DNA (Figure 1G). Although the spatial resolution of qRamanomics is lower compared with confocal microscopy (Figure 1H), the analysis demonstrates that qRamanomics allows quantitative, label-free, multi-component analysis of 3D PHH including components that are not readily visualized by label-dependent confocal microscopy. Hence, the technology can serve as a tool for evaluating qualities in 3D organoids such as spatial distribution, metabolic maturity, and response to diverse agents. The qRamanomics studies described here were done on fixed tissue, but the technique could be expanded to chemometric measurements in living organoid tissue with the goal of providing a tracking tool for organoid cultures. Toward this goal, further advances in sensitivity and data deconvolution are required.

### Establishment of multi-component qRamanomics as a benchmarking tool for 3D liver representations: Comparison of primary human hepatocyte spheroids with iPSC-derived hepatic organoids

We next explored the potential of qRamanomics for determining quantitative and qualitative differences within 3D PHH and 3D iHLC batches, followed by a comparison between them.

**Figure 2:**
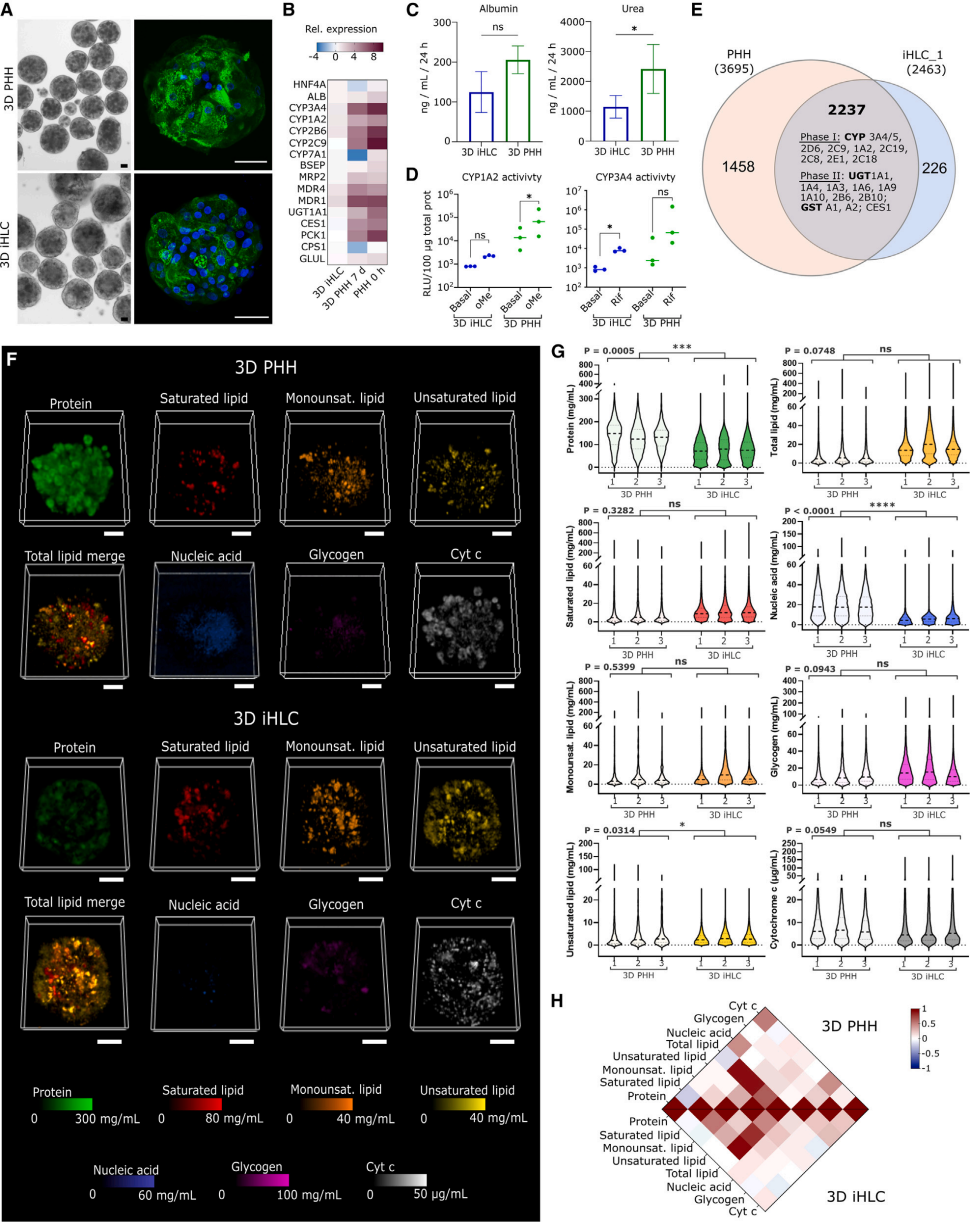
qRamanomics enables quantitative high-content composition comparison between primary human hepatocyte spheroids and induced hepatocyte-like cell organoids (A) Representative bright-field and immunofluorescence images of 3D PHHs and 3D iHLCs (green, albumin; blue, nuclei). Scale bar, 50 μm. (B) Heatmap shows mean relative expression of selected hepatocyte-specific genes given as log_2_. iHLC organoids from iPSC line WTSIi028-A (iHLC_3) were used for the normalization. (C) Secretion of albumin and urea in PHH and iHLC organoids. (p = 0.059 for albumin secretion, p = 0.0386 for urea secretion.) (D) Basal and induced cytochrome P450 activity. Data represent mean for three donors of 3D PHHs and three cell lines of 3D iHLCs. (CYP1A2 activity in iHLC, p = 0.057, in PHH p = 0.039; CYP3A4 activity in iHLC, p = 0.0266, in PHH p = 0.3902.) (E) Venn diagram of proteins detected in PHH (n = 1 donor) and iHLC organoids (n = 1 cell line). (F) High-content quantitative Ramanomic imaging reveals distribution of molecular content throughout 3D PHH spheroids from a single donor (n = 3) and 3D iHLC organoids (n = 3). Scale bars, 50 μm. (G) Inter-spheroid/organoid repeatability within and between specimen groups. Nested t test revealed significant differences in protein, unsaturated lipid, and nucleic acid content between 3D PHH and 3D iHLC samples. (p values are indicated on the figure itself.) (H) Pearson’s correlation chemometric heatmaps illustrate co-localization of various molecular components in 3D PHH and iHLC.

For validating and comparing hepatic 3D representations, 3D PHH of standardized size were created by hepatocyte aggregation in microwells^22^ (Figure 2A). Human 3D iHLC were generated by a modified last-generation differentiation protocol, which enables production of highly standardized organoids with significant metabolic maturity^23^^,^^24^ (Figures 2A–2D). Relative expression of hepatocyte-specific genes as measured by qRT-PCR indicated the expression of hepatocyte-specific genes such as *HNF4A*, *CYP3A4*, *CYP1A2*, *CYP2B6*, and *MDR1*,*4* in the 3D iHLC, albeit at significantly reduced levels compared with 3D PHH (Figure 2B). 3D iHLC faithfully showed hepatocyte-specific functionality, including albumin production (n = 3), and urea secretion at ∼50% lower levels, compared with 3D PHH cultures (Figure 2C). 3D iHLC showed CYP1A2 and CYP3A4 enzymatic activity both basal and induced by omeprazole and rifampicin, respectively, albeit at lower levels than in 3D PHH (Figure 2D). A comprehensive proteomics analysis further confirmed the presence of most phase I enzymes, as well as phase II enzymes and transporters within 2,237 joint protein signatures in both 3D liver representations (Figures 2E and S2F), while differences were also present as seen in 1,458 (3D PHH) and 226 (3D iHLC) non-overlapping protein signatures (Figure 2E). Taken together, our spheroid/organoid protocols allowed generation of metabolically active 3D hepatic representations from both iHLC and PHH suitable for the testing of qRamanomics as a benchmarking tool.

For comparing 3D iHLC vs. 3D PHH by qRamanomics, Raman data were pre-processed and deconvoluted to quantitatively map the spatial distribution of the aforementioned deconvoluted biomolecule spectra in both 3D PHH and 3D iHLC (Figure 2F). Despite a modest variance in spheroid and organoid size (Figure S2E, coefficient of variation from 63% to 82% for 3D iHLC and from 64% to 90% for 3D PHH), component quantity, and distribution remained consistent within each group, revealing intra-group homogeneity (Figure 2G), as well as statistically significant inter-group differences between 3D PHH and 3D iHLC (Figures 2G and S3). Noteworthy statistical differences between 3D PHH and 3D iHLC were seen in protein amounts, nucleic acid, and unsaturated lipids, short-listing those markers as qualifiers for benchmarking tools. Total protein concentrations, a marker for functional activity, were subsequently confirmed by UV spectroscopy and proteomics (Figures S2D and S2F).

The potential of the qRamanomics technology was further revealed when component spectra were correlated with each other and between 3D PHH and 3D iHLC in correlative heatmaps (Figure 2H). In the heatmaps, data are shown as Pearson’s correlation, measuring individually the linear correlation between two sets of components in all voxels. Hence, red color on the map indicates predominant co-localization of two classes of biomolecules in scanned voxels, white indicates that there is a random correlation between two components in the measured voxels, and blue indicates mutually exclusive localization of components. Importantly, the heatmap illustrates in detail differences between 3D PHH and 3D iHLC. Overall, the heatmap shows similar lipid distribution in 3D PHH and 3D iHLC relative to proteins (a conventional standard for normalization in traditional bulk biochemical assays). However, saturated lipids were to a higher degree co-localized with proteins in 3D iHLC compared with 3D PHH. In contrast, nucleic acids more frequently co-localized with proteins in 3D PHH compared with 3D iHLC. Hepatocyte polyploidy is hypothesized to be an adaptive mechanism for enhanced metabolism and protein synthesis as well as a mechanism to increase tolerance to the genomic stress and apoptotic signals.^25^^,^^26^ Higher number of binuclear cells and more active protein synthesis in 3D PHH could explain detected differences.^25^ A further noteworthy difference is found in the Cyt *c*/protein co-localization as well as the Cyt *c*/nucleic acid co-localization, which is remarkably higher in 3D PHH than in 3D iHLC, while the total Cyt *c* content was at the same level in both groups (Figure 2G). Cyt *c* is involved in the electron transport chain in the mitochondrial inner membrane. Oxidative phosphorylation is characteristic for metabolism of differentiated and adult cells,^27^ and proteomics confirmed the presence of citric cycle enzymes and mitochondrial respiratory chain components in 3D iHLC (Figure S2D). Despite these differences, the heatmap also revealed similar patterns of glycogen/protein co-distribution, glycogen being an important hallmark of hepatocyte functionality and indicative of the maintenance of glucose homeostasis.

In summary, multi-component chemometric qRamanomics on 3D hepatic spheroid/organoids enabled a direct, data-rich, label-free characterization of 3D tissue representations with foreseeable utility as a corroborative quality control tool. Further analysis can be expanded toward directly linking each multi-component voxel to subcellular structures^28^ and hence adding further granularity in the analysis.

### qRamanomics reveals compositional phenotypic changes in 3D liver representations in response to drug exposure

Next, we applied the platform to interrogate 3D liver models with a panel of drugs with reported impact on hepatocytes (Figure S4A). Drug-induced lipid accumulation in the mouse liver, using semi-quantitative coherent anti-Stokes Raman spectroscopy, has been previously reported.^29^ However, multi-component quantitative chemometric phenotyping of liver spheroids/organoids has hitherto not been possible.

**Figure 3:**
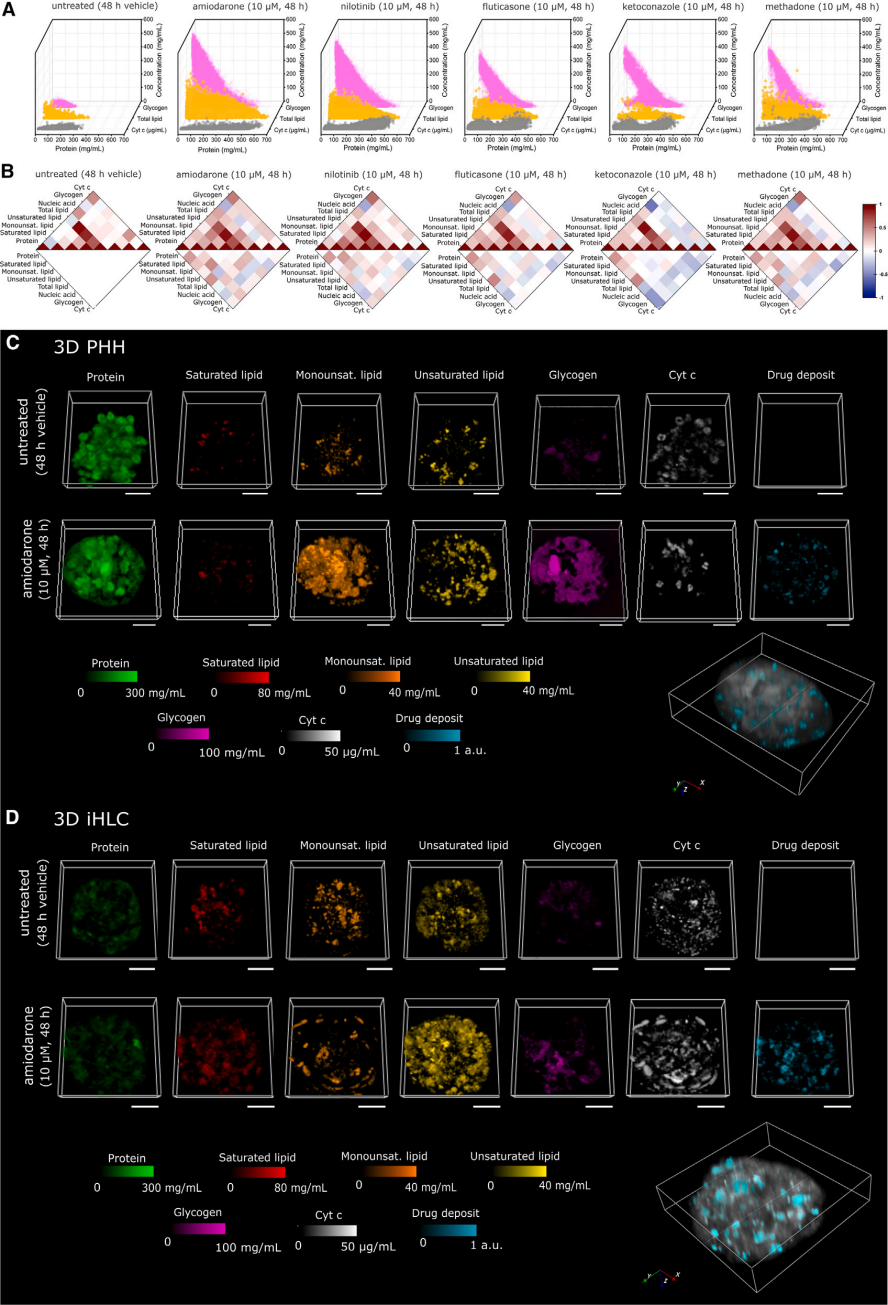
qRamanomics reveals high-content compositional phenotypic changes in response to drug treatment (A) Frequency distribution plots of total lipid, glycogen, and cytochrome *c* vs. protein content for representative drug-treated samples. High-content chemometric profiling of individual control (48 h of vehicle treatment). (B) Drug-specific changes in compositional phenotype for drug-treated 3D PHHs by quantitative high-content correlation analysis (n = 4 spheroids per group). Top half of plots shows Pearson’s correlation and bottom half shows absolute difference compared with untreated control. (C and D) untreated and amiodarone-treated (10 μM, 48 h) 3D PHH spheroids (C) and iHLC organoids (D). Amiodarone induces significant measurable changes in biomolecular composition of both 3D liver representations. Scale bars, 50 μm. 3D renderings of amiodarone and metabolites deposits (shown in cyan) detected in PHH spheroids and iHLC organoids. The biomolecular matrix (i.e., protein, total lipid, and glycogen) was combined and is shown in gray (arbitrary intensity units) to reveal clear distinction between endogenous biomolecules and xenobiotic compounds present in specimens.

3D PHH and 3D iHLC were exposed for 48 h to 10 μM amiodarone (an antiarrhythmic drug), nilotinib (a BCR-ABL tyrosine kinase inhibitor), fluticasone propionate (a corticosteroid hormone receptor antagonist), ketoconazole (an antifungal agent preventing the synthesis of ergosterol), or methadone (an opioid analgesic), and subsequently analyzed by qRamanomics and conventional assays (Figures 3, S4, and S5). The interrogation of neratinib-treated organoids required a 785-nm excitation laser (see below) and hence a different set of reference spectra. It was therefore not included in this part of the study.

Waterfall plots (Figure 3A) of control and drug-treated 3D PHH revealed substantial changes in drug-treated spheroids. Strikingly, in the treatment groups there is an increase in number and concentration of Cyt *c*, total lipid, and glycogen voxels, and in particular the presence of voxels of these three biomolecules in areas of high protein concentration (>300 mg/mL). The protein concentration in the cytoplasm has been estimated to be overall 100 mg/mL while in the rat liver it reaches 310 mg/mL, covering 20%–30% of the cell volume^30^ (see untreated control group with a protein concentration of <300 mg/mL). Hence, the presence of Cyt *c* voxels containing protein concentrations of up to 600 mg/mL, as in the amiodarone treatment group (up to 500 mg/mL in other treatment groups), indicates the presence of condensed protein states. The distribution of lipid and glycogen followed the opposite pattern: voxels with the highest concentration of these biomolecules did not contain proteins or contained them in low concentration, indicating segregated accumulations.

Applying a heatmap that shows in the upper part the Pearson’s correlation between biomolecules within a 3D spheroid in each group and in the lower part the absolute difference compared with the untreated control, we gained an overview of tell-tale drug-induced alterations of biomolecules (Figure 3B). We observed a drug-specific increase in total lipid/protein as already visualized in a waterfall graph, being most significant in the amiodarone and nilotinib groups. More detailed analysis revealed marked changes in protein/saturated, monounsaturated, and unsaturated lipid co-localization, in particular in the amiodarone, nilotinib, and methadone treatment groups^31^^,^^32^ (Figure 3B). Lipid accumulation is a sign for drug-induced toxicity often associated systemic metabolic dysfunction, while the ratio of saturated-to-unsaturated fatty acids in cells is an important context-dependent determinant of cell viability.^33^ The reduced viability of amiodarone-exposed spheroids was confirmed by a decrease in ATP content and albumin production correspondingly in 3D PHH and 3D iHLC (Figures S4B and S4C).

Elevated levels of glycogen that delocalize with protein-containing voxels emerged as a further noteworthy observation in all treatment groups. Hepatic carbohydrate metabolism, including glycogen accumulation, can be impaired by pathological processes and xenobiotic exposure.^34^^,^^35^^,^^36^^,^^37^^,^^38^ For instance, a recent study^37^ showed that glycogenosis (excessive glycogen accumulation) is common for both pediatric and adult patients with non-alcoholic fatty liver disease and is indirectly correlated with greater cell injury. There are currently only a few reports on drug-induced changes of subcellular hepatic glycogen accumulation,^34^^,^^36^ possibly due to methodological difficulties of quantitative evaluation of glycogen in subcellular compartments. Excessive glycogen accumulation beyond normal levels could point to pathological changes in hepatocyte metabolism accompanying or causing hepatotoxic effects. Although currently lacking definitive evidence, this hypothesis is testable in future studies using our proposed qRamanomics methodology.

3D PHH treated with a subtoxic dose of ketoconazole (10 μM) provided a particularly interesting case. Ketoconazole may cause liver damage in a dose-dependent manner.^39^ We observed significantly elevated glycogen and Cyt *c* in the treatment group, concomitant with a marked uncoupling of Cyt *c* with protein-containing voxels (Figure 3B). There is no direct explanation for these phenomena, but further research can probe a link between ketoconazole-induced activation of NRF2 and enhanced glycogenosis^40^ while Cyt *c*/protein uncoupling may be linked to a mitophagy-mediated mitochondrial dysfunction promoted by ketoconazole.^41^

We next compared the alterations after exposure to 10 μM amiodarone—a commonly used model hepatotoxic drug^42^ leading to microvesicular steatosis and phospholipidosis^43^^,^^44^—in both 3D PHH and 3D iHLC (Figures S4D and S4E). Drug-induced changes in both liver models were similar except for levels of glycogen accumulation (lower in 3D iHLC as compared with 3D PHH), suggesting that drug responses in 3D iHLC closely mimicked those in 3D PHH. Accordingly, qRamanomics reveals changes of monounsaturated and unsaturated lipids in the amiodarone-treated 3D PHH and 3D iHLC (Figures 3C and 3D) with a consistent concentration- and time-dependent increase of lipid-containing voxel frequency (Figure S4F). Among all tested drugs, amiodarone treatment induced the most significant changes in glycogen (increase) in both groups, and a decrease of Cyt *c* in 3D PHH (Figures 3C and 3D). The decreased Cyt *c* content (Figure 3A) may be a consequence of previously described amiodarone-induced mitochondrial toxicity and an inhibition of the mitochondrial respiratory chain.^45^ To the best of our knowledge, amiodarone-induced glycogenosis has not yet been reported.

### Correlative *in situ* detection of xenobiotic deposits in 3D PHH and 3D iHLC

**Figure 4:**
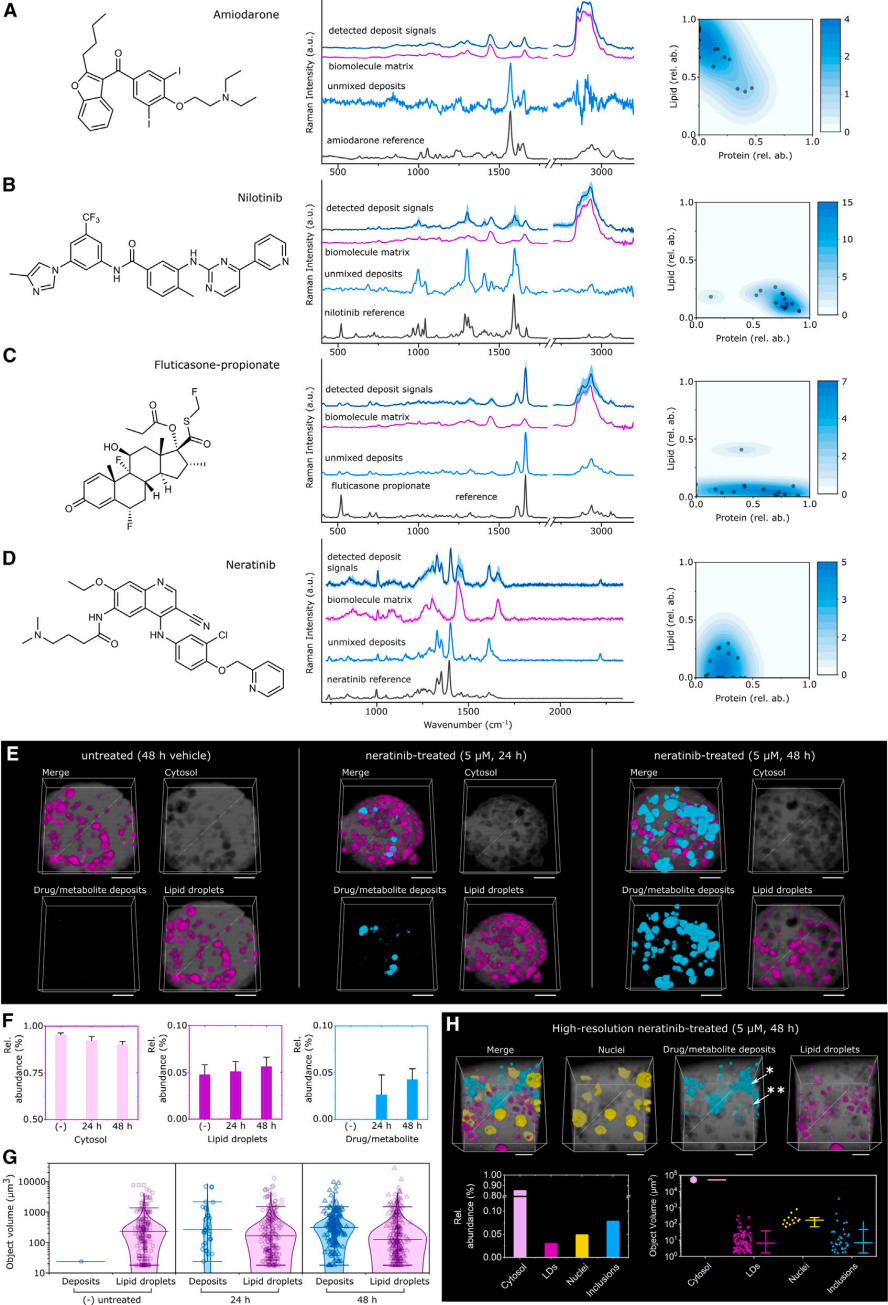
Drug and drug metabolite deposits were detected within lysosomes yielding altered Raman spectral signals as compared with parent drug for drug-treated 3D iHLC organoids (A–D) Chemical formulas for drugs whereby detected deposit signals were extracted from intracellular drug and/or metabolite aggregate regions of interest (five deposits from n = 3 3D iHLCs for each treatment group) for (A) amiodarone, (B) nilotinib, (C) fluticasone propionate, and (D) neratinib-treated 3D iHLC organoids. Spectral difference between unmixed deposits and parent drug reference signals suggests metabolism of compounds. (E and F) (E) qVRI reveals distribution of drug/metabolite (cyan) and lipid droplets (magenta) for neratinib-treated 3D PHHs (scale bars, 50 μm). (F) Relative volumetric abundance for each component. (G) Particle size distribution analysis of drug/metabolite deposits (inclusions) within drug-treated and control specimens (n = 3 organoids per group). (H) High-resolution 3D Raman chemical imaging suggests neratinib accumulation in lysosomes (∗∗) and excretion to bile canaliculi (∗). The mean and error bars are plotted adjacent to the data points to aid clarity. Scale bars, 10 μm.

Following the characterization of drug alterations in 3D liver representations, we explored the utility of RSI for measuring contextual drug and metabolite accumulation in organoids. This is done by setting the unique molecular vibrations arising from xenobiotics in context with the aforementioned deconvoluted and quantifiable biomolecule spectra. Here we report the first spectroscopic evidence of xenobiotic deposits of amiodarone and fluticasone detectable by RSI within 3D PHH and 3D iHLC, along with evidence of nilotinib accumulation (Figures 3C, 3D, and 4A–4D), whereby we used the aforementioned qRamanomics dataset for multi-variant voxel associations. For neratinib, the 532-nm excitation laser is not suitable due to autofluorescence of the compound, and instead a 785-nm laser had to be applied. No deposits were detectable with our setting in the ketoconazole or methadone treatment groups (Figure S5). Kernel-density probability estimates for unmixed deposit spectra revealed that each detectable xenobiotic exhibited unique drug-biomolecule complexes as determined by the relative abundance of lipid/protein in each voxel (Figures 4A–4D). This allowed us to elucidate xenobiotic-cell interactions in 3D iHLC, showing for example that amiodarone (a known lipophilic drug with a long half-life of >60 days^39^) would faithfully accumulate in lipid-rich subcellular voxels, while the nilotinib spectra (also being lipophilic but having a considerably shorter half-life of 17 h^46^) would be detected in protein-rich voxels, indicating metabolic processing. Neratinib spectra, in contrast, accumulated in voxels that are neither protein nor lipid rich. Subtracting the biomolecule signals from detected deposits revealed spectra for “pure” unmixed deposits of xenobiotics. Comparison of unmixed deposit spectra with parent drug reference spectra revealed differences attributable to changes in molecular bond/structure of the molecule, as was the case for nilotinib, fluticasone propionate, and neratinib. These spectroscopic changes could be evidence of drug metabolism as previously described for neratinib in cancer cells.^13^ Hence, the conjunction of changes in the spectra of nilotinib and the accumulation of the xenobiotic in the protein compartment together with its known short half-life could be evidence for an active, CYP3A4 dependent drug metabolism of nilotinib in 3D iHLC.

We next investigated in further detail in 3D liver representations neratinib and its metabolites, which interestingly showed an accumulation with neither lipid nor protein voxels. Owing to the wavelength of the fluorescence signals of neratinib, Raman data were acquired with a 785-nm excitation laser (instead of 532 nm as previously described) and quantified using qVRI, a previously introduced quantification technology.^14^ The presence of neratinib and its metabolites was independently confirmed in both 3D PHH and 3D iHLC pellets and supernatant by high-performance liquid chromatography-mass spectrometry (LC-MS) (Figure S6F and S7). High-resolution qVRI imaging enabled clear distinction of nuclei and verified particle size distribution measurements (Figure 4H). Raman image segmentation using Ilastik software^47^ allowed 3D visualization (Figure 4E) and volumetric measurements of continuous neratinib metabolite deposits (Figure 4F), which were smaller than the volume of typical hepatocytes (<3,500 μm^3^), indicative of intracellular localization (Figure 4G). 3D visualization suggested that larger deposits represent metabolites excreted into the bile canaliculi-like structures, observed in both 3D PHH and 3D iHLC (Figure S6E); however, intracellular deposits required further clarification.

The strong fluorescence signal of neratinib and its metabolites allowed an independent confirmation by confocal imaging (Figure S6C) which, unlike RSI, cannot reveal chemical composition of deposits and is applicable only to a limited number of drugs with strong fluorescence (for example, only neratinib fulfilled these criteria within our panel). For this drug, confocal microscopy revealed an accumulation of a strong fluorescent signal in lysosomes as marked by the lysosomal membrane protein LAMP1 after neratinib exposure (Figure S6D). Therefore, lysosomal accumulation is a likely explanation for the localized intracellular neratinib puncta that were observed by RSI in the 3D iHLC. Indeed, lysosomal accumulation and phospholipidosis (Figures S6A and S6B) are proposed as possible mechanisms of neratinib-induced hepatic toxicity.^48^

In summary, for drugs identifiable by RSI, a label-free spatial and temporal “snapshot” of their presence and processing is now available, comprising factors including drug/metabolite abundance and spatial distribution in the context of drug-induced multi-factorial changes in 3D hepatic representations.

## Discussion

The role of localized concentrations and biomolecular density at subcellular levels is gaining attention as a relevant phenotypic parameter for cell-type-specific functions^49^^,^^50^ and can be indicative, for example, of the maturation state of cells and their response to xenobiotic exposures.^49^

In this study, we developed a robust data-processing and calibration pipeline, qRamanomics, that allows label-free, multi-factorial, chemometric phenotyping of 3D biospecimens using RSI. Unlike conventional fluorescence imaging tools that are mostly optimized for proteins, the qRamanomics framework described here enables direct quantitative *in situ* classification of a broad range of biomolecules, including different classes of lipids, Cyt *c*, proteins, nucleic acids, glycogen, vitamins, and selected xenobiotics. The technology therefore promises multiple practical applications in organoid research and development.

Unlike prior methods,^5^ we used a liquid water Raman signal for the normalization of all analyte Raman signals, enabling direct quantification of biomolecules. The underlying assumption was that the water content remains constant within the excitation voxel sampling volume (∼5 fL) across linear quantitation ranges of biomolecule concentration. When an analyte concentration exceeds aqueous solubility, phase separation may occur and, in this case, the quantitative accuracy of the model becomes difficult to verify with this current iteration of calibration technology. Nevertheless, measurements beyond the upper range of biomolecule calibration standard phantoms are presented under the assumption that linearity remains constant. A similar approach has been recently published for stimulated Raman scattering microscopy; however, it was limited to evaluation of protein and lipid concentration.^51^ Further development of instrumental calibration technology is still necessary to facilitate inter-laboratory comparisons of Raman cytometry data and for harmonizing qRamanomics at the global level.^52^ Moreover, adding additional spectral signatures to the analysis and establishing a library of tissue phantoms for a broader range of biomolecules will further expand the application of qRamanomics. For instance, by visualizing retinoids in combination with lipids, our approach may facilitate studying 3D liver fibrosis models. Moreover, qRamanomics is well suited as a bioanalytical tool to explore drug-induced quantitative chemometric alterations in 3D liver representations. Using qRamanomics we were able to quantitatively describe drug-induced alterations of biomolecular markers in 3D PHH and 3D iHLC. While the mechanistic link between drug-induced toxicity and intracellular drug-lipid accumulation, as well as glycogen accumulation, is currently not well understood, qRamanomics paves the way for analyzing adaptive and pathological changes in hepatocyte metabolism in response to xenobiotics. This analysis is further enhanced by the ability to visualize and analyze drug metabolism and deposition of selected xenobiotics *in situ*.

qRamanomics provides a solution to address quantitative in-depth chemometric characterization of 3D tissue representations with cellular and subcellular resolution. This should enable detailed and label-free interrogation of 3D tissue representations that is complementary to existing imaging technologies.

### Limitations of the study

The calibrated bioanalytical platform for quantitative chemometric phenotyping, described in this study, currently has the following limitations. First, the depth of reliable data acquisition was limited to 50 μm in the z plane, therefore representing only a segment of organoids. Tissue penetration may be further increased by applying tissue-clearing methodology.^2^ Second, the current sensitivity of the technology requires long data-acquisition times and hence requires fixed tissues. Steps are being taken to advance the technology toward live tracking of 3D biospecimens. This may be achieved by a combination of an increased sensitivity of the hardware, a reduction of the 3D tracking area by applying light-sheet technologies, and improved spectral pattern recognition by implementing machine-learning algorithms. Third, a methodology for correlating the biomolecular composition and density of analyzed voxels to subcellular structures would help to extract more information from the spectra, including the distinguishing of nucleic acid types (RNA vs. DNA). This could, for example, be attempted by identifying organelle-specific fingerprints.^53^ These limitations motivate future work to further develop the platform. Nonetheless, the qRamanomics platform, as described here, opens the doors of the unadulterated biomolecular milieu within living cells and tissues to scientific interrogation.

## STAR★Methods

### Key resources table

**Table:** 

REAGENT or RESOURCE	SOURCE	IDENTIFIER
**Antibodies**		

Rabbit Human Serum Albumin Polyclonal antibody	Abcam	Cat# ab2406; RRID: AB_303048
Goat ZO-1 Polyclonal Antibody	Thermo Fisher Scientific	Cat# PA5-19090; RRID: AB_10988668
Mouse CYP3A4 Monoclonal Antibody (Clone 3H8)	Thermo Fisher Scientific	Cat# MA5-17064; RRID: AB_2538535
Mouse Anti-MRP2 Monoclonal Antibody (Clone M2 III-6)	Abcam	Cat# ab3373; RRID: AB_303751
Alexa Fluor 488-AffiniPure Donkey Anti-Goat IgG (H+L) (min X Ck,GP,Sy Hms,Hrs,Hu,Ms,Rb,Rat Sr Prot)	Jackson ImmunoResearch Labs	Cat# 705-545-147; RRID: AB_2336933
Cy3-AffiniPure Donkey Anti-Rabbit IgG (H+L) (min X Bov,Ck,Gt,GP,Sy Hms,Hrs,Hu,Ms,Rat,Shp Sr Prot)	Jackson ImmunoResearch Labs	Cat# 711-165-152; RRID: AB_2307443
Alexa Fluor® 647 AffiniPure Donkey Anti-Mouse IgG (H+L)	Jackson ImmunoResearch Labs	Cat# 715-605-150; RRID: AB_2340862

**Biological samples**		

Cryopreserved primary human hepatocytes	Gibco	Cat# HMCPSQ; lot HU8339-A
Cryopreserved primary human hepatocytes	Lonza	Cat# HUCPG; lot HUM180201A
Cryopreserved primary human hepatocytes	Gibco	Cat# HMCPMS; lot HU8287
XTreme 200 POOL HUMAN LIVER MICROSOMES	Tebubio	Cat#H2630; Lot nr: 171008

**Chemicals**, **peptides**, **and recombinant proteins**		

Neratinib	Selleckchem	Cat# S2150
Nilotinib	Selleckchem	Cat# S1033
Ketoconazole	Selleckchem	Cat# S1353
Amiodarone HCl	Selleckchem	Cat# S1979
Fluticasone propionate	Selleckchem	Cat# S1992
Methadone stock solution	This paper	n/a

**Critical commercial assays**		

CellTiter-Glo® 3D Cell Viability Assay	Promega	Cat# G9681
P450-Glo™ Assay with Luciferin-IPA	Promega	Cat# V9001
P450-Glo™ CYP1A2 Assay	Promega	Cat# V8771
HCS LipidTOX™ Red neutral lipid stain	Thermo Fisher Scientific	Cat# H34476
Human Albumin ELISA Quantitation Set	Bethyl Laboratories	Cat# E88-129

**Experimental models**: **Cell lines**		

Induced pluripotent stem cell WTC-11	Coriell Institute for Medical Research	RRID:CVCL_Y803
Induced pluripotent stem cell WTSIi013-A	Wellcome Trust Sanger Institute	RRID:CVCL_AE60
Induced pluripotent stem cell WTSIi028-A	Wellcome Trust Sanger Institute	RRID:CVCL_AI14

**Software and algorithms**		

Fiji	Schneider et al.^7^	https://imagej.nih.gov/ij/
PEAKS X+ software version 10.5	Bioinformatics Solutions	https://www.bioinfor.com/peaks-studio/
GraphPad PRISM 7	GraphPad Software Inc.	
All original code has been deposited at GitHub repository and is publicly available via Zenodo: https://doi.org/10.5281/zenodo.7628912	Developed in-house during the project	https://doi.org/10.5281/zenodo.7628912

### Resource availability

#### Lead contact

Further information and requests for resources and reagents should be directed to and will be fulfilled by the lead contact, Molly M. Stevens (m.stevens@imperial.ac.uk).

#### Materials availability

This study did not generate new unique reagents. Unique code was generated, as detailed below.

### Experimental model and subject details

#### Primary human hepatocytes culture

Cryopreserved primary human hepatocytes (Gibco, catalogue no. HMCPSQ, lot HU8339-A, female (referred in the study as PHH_1), Lonza, catalogue no. HUCPG, lot HUM180201A, male (referred in the study as PHH_2) and Gibco, catalogue no. HMCPMS, lot HU8287, female (referred in the study as PHH_3)) were thawed in the Hepatocytes thaw media (Gibco, catalogue no. CM7500) according to manufacturer’s protocol. Moreover, one sample of PHH (donor 73 years old, male) was obtained from the Department of Clinical Science, Intervention and Technology (CLINTEC), Division of Transplantation Surgery, Karolinska Institutet (Stockholm, Sweden). The regional committee for medical and health research ethics in Norway approved the use of human material (REK 50786). Uniform PHH spheroids were created by aggregation in ultra-low attachment micro-wells (Corning, catalogue no. 4440) or in house-made agarose microwells - a format in which PHH showed stable functionality over at least 7 days as described before^22^ (Figure S2G). Briefly, cells were plated into microwells at the concentration of 1000 viable cells per microwell and were centrifuged at 100 g for 2 min. For the first 3 days PHH were cultured in the Williams E medium (Thermo Fisher Scientific, catalogue no. A1217601) supplemented with 7 % FBS (Thermo Fisher Scientific, catalogue no. 41400045), 2 mM L-glutamine (Thermo Fisher Scientific, catalogue no. 35050038), 10 μg/ml insulin, 5.5 μg/ml transferrin, 6.7 ng/ml sodium selenite (Thermo Fisher Scientific, catalogue no. 41400045) and 0.1 μM dexamethasone (Sigma Aldrich, catalogue no. D4902). From day 4 onwards, the FBS concentration was gradually decreased till 1 % (v/v). Spheroids were maintained in serum free media from day 7 for up to 2 weeks.

Human induced pluripotent stem cells. Human induced pluripotent stem cells (iHLC_1: WTC-11, Coriell Institute for Medical Research; iHLC_2: WTSIi013-A and iHLC_3: WTSIi028-A, Wellcome Trust Sanger Institute) were cultured in E8 media (Thermo Fisher Scientific, catalogue no. A1517001) on plates coated with 0.1 % (v/v) Geltrex (Thermo Fisher Scientific, catalogue no. A1413201) in a humidified 37°C, 5% CO_2_ incubator. Cells were passaged using 0.5mM EDTA (Thermo Fisher Scientific) in DPBS and replated as small clumps at a dilution 1:3-1:5. Quality control, performed after thawing of cells, included flow cytometry, qPCR, immunofluorescent imaging for pluripotency markers and karyotyping.

#### Differentiation of human iPSC-derived 3D iHLC

Organoids formed by hepatocyte-like cells (3D iHLC) were generated using modification of previously published protocols.^23^^,^^24^ First, iPSC were differentiated toward definitive endoderm in IMDM/F12 media containing 1 % (v/v) lipid concentrate (Thermo Fisher Scientific, catalogue no. 11905031), 100 μg/ml transferrin, 3 μM CHIR99021 (Tocris Bioscience, catalogue no. 4423), 50 nM PI-103 (Tocris Bioscience, catalogue no. 2930) and 100 ng/ml activin A (Peprotech, catalogue no. 120-14P) for 24 h and 100 ng/ml activin A for subsequent 48 h. The definitive endoderm cells were treated with 10 ng/mL FGF2 (Peprotech, catalogue no. 100-18B) and 20 ng/mL BMP4 (Peprotech, catalogue no. 120-05) in IMDM/F12 medium supplemented with 1% (v/v) N-2 (Thermo Fisher Scientific, catalogue no. 17502-048), 1% (v/v) B-27 minus vitamin A (Thermo Fisher Scientific, catalogue no. 12587010) and 1% (v/v) lipid concentrate, then with 5 μM A8301 (Stem Cell Technologies, catalogue no. 72022), 20 ng/mL HGF (Peprotech, catalogue no. 100-39H), 20 ng/mL BMP4, 1% (v/v) B-27 with vitamin A for 3 more days and with 25 ng/mL HGF, 1% (v/v) DMSO for another 5 days. At day 12, cells were detached and aggregated in the agarose U bottom microwells in the presence of 25 ng/mL HGF, 0.1 μM Dexamethasone, 0.5% (v/v) ITS, 0.1% (v/v) lipids concentrate, 100 μM Ascorbic acid-2 phosphate (AAP), 1% (v/v) B-27 (without vitamin A) and 1% (v/v) N-2. After formation of spheroids at day 13, media was replaced with William’s E media, supplemented with 5% (v/v) FBS, 20 ng/ml HGF and 10 ng/ml oncostatin M (Peprotech, catalogue no. 300-10), 1% (v/v) ITS, 100 μM AAP, 0.1 μM Dexamethasone. For further maturation, organoids were cultured in microwells in William's E media, supplemented with 1% (v/v) ITS, 0.1 μM Dexamethasone, 20 ng/ml Oncostatin M and 1% (v/v) MEM Non-Essential Amino Acids Solution (Thermo Fisher Scientific, catalogue no. 11140050), for another 10 days. At day 18, organoids were additionally incubated for 1 h in the same medium supplemented with 5% (v/v) of Geltrex.

### Method details

#### Biomolecular tissue phantoms as 3D quantitative calibration standards

Tissue phantoms are artificial structures that mimic tissue-like properties, commonly mechanical or optical, in a reliable and reproducible way, widely used to test instrumental performance. Aqueous-based gels containing known concentrations of the most abundant cellular biomolecules were formulated in PBS. Bovine serum albumin (BSA) (Sigma Aldrich) was selected as protein representative due to its inexpensiveness, high water solubility and miscibility with other biomolecules. Synthetic high-density lipoproteins (sHDL) composed of 22A peptide (PVLDLFRELLNELLEALKQKLK) and DPPC or POPC were prepared by a co-lyophilization procedure. Briefly, peptide and phospholipid were dissolved in glacial acetic acid, mixed at 1:2 wt/wt ratio, and lyophilized overnight. The powder was rehydrated with water to make 80 mg mL^−1^ (based on DPPC concentration) sHDL or 50 mg mL^−1^ (based on POPC concentration) and thermocycled. For DPPC the procedure was between 50°C (10 min) and room temperature (10 min) thrice and for POPC it was room temperature (10 min) and ice bath (10 min) thrice to facilitate sHDL formation. The resulting HDL complexes were diluted to 1 mg mL^−1^ (based on peptide concentration) with water and analyzed by gel permeation chromatography (GPC) for purity using 7.8 mm × 30 cm Tosoh TSK gel G3000SWxl column (Tosoh Bioscience) with 1 mL min^−1^ flow rate (PBS pH 7.4). Free peptide and sHDL peaks were detected at 220 nm. The sHDL hydrodynamic diameters were determined in water at 1 mg mL^−1^ by dynamic light scattering (DLS) using a Zetasizer Nano ZSP, Malvern Instruments (Westborough, MA). The volume intensity average values (±SD) were reported.

Intralipid 20 % (Sigma-Aldrich, I141-100ML) was initially employed as lipid reference but was excluded from the model due to quantitative inconsistencies resulting from larger particle size of the emulsion formulation as compared with HDLs. In the case of nucleic acids, commercially available salmon testes DNA (Sigma-Aldrich, St. Louis, MO, USA) was diluted in deionized water at 50°C and pH 8 prior to gelation. BSA and IL were also subjected to dynamic light scattering (DLS) analysis (Zetasizer Nanoseries, Malvern Instruments, Ltd) to ensure homogeneity and discard molecular aggregation. Cytochrome c (Cyt c) signals were included in the quantitative model (after detection in biological specimens) by dissolving 100 μg/mL cytochrome c (Sigma-Aldrich, C3131-50MG) in PBS to elucidate the concentration-dependent signal intensity (a.u. per μg/mL). Cyt c signals are commonly reported in various biospecimens and yield signals ∼1,000-fold stronger than other biomolecules due to resonance Raman effect when excited with 532 nm light.^1^^,^^3^^,^^4^

#### Raman spectral acquisition

All spectra were acquired using a WITec alpha 300R + Raman confocal microspectrometer (Ulm, Germany) equipped with a piezoelectric stage (UHTS 300, WITec, GmbH.), 50X air objective lens (Zeiss EC EPIPLAN, N.A. = 0.75), 63x water immersive objective lens (Zeiss W Plan Apochromat 63X, N.A = 1), green solid-state excitation laser (λ = 532 nm, 32 mW, WITec, GmbH.) and an imaging spectrograph (Newton, Andor Technology Ltd. UK) equipped with a 600 groove/mm grating and a thermoelectrically cooled (60°C) charged-coupled detector (CCD) optically connected to the objective through a 10 μm diameter single mode silica fiber-optic cable. This setup enabled acquisition of spectral data across a wavenumber range from 0-3600 cm^−1^. Raman depth scans of tissue phantom calibration set were performed by first locating the highest SNR laser focal plane for sample excitation at the tissue phantom surface, followed by continuous scanning data acquisition through the depth profile of interest at each x-z position. Similarly, z-stacks for quantitative chemometric profiling were acquired sequentially with 10 μm step size, starting from the sample surface. Each stack was acquired in raster patter as a 100∗100 x-y 2D hyperspectral image with 2 μm spatial resolution. In all cases, the excitation laser intensity was kept constant between sample scans, as well as integration time of 0.25 s.

#### Three-dimensional Raman chemical imaging of iHLC organoids and PHH spheroids

Upon maturation, all specimens were fixed with paraformaldehyde (PFA) 4 % (v/v) and embedded in 1 % (w/v) agarose (Sigma-Aldrich, A0701-25G) microwells with phosphate-buffered saline (PBS) (pH 7.4, Gibco) for immobilization in hydrated state during chemical imaging.^10^^,^^11^^,^^12^^,^^13^ Using 63X water immersion objective, 50 μm deep Z-stack scans were acquired with X-Y step size of 2 μm, Z step size of 10 μm, and integration time of 0.25 s per voxel. Total image size was dependant on each organoids’ physical dimensions, ranging from 100-180 ∗ 150-250 pixels, but keeping X-Y and Z resolution at 2 and 10 μm respectively. Additionally, organoid image stacks were subjected to SNR thresholding to remove all voxels where maxima in the high wavenumber region (2800 – 3100 cm^-1^) were less than 10-fold the standard deviation observed across the baseline of Raman-silent region (2200 – 2600 cm^-1^) of same spectra to ensure only high SNR spectral data was included in the quantitative analysis. Each hyperspectral image in every stack was preprocessed as described and run through the selected statistical unmixing algorithm. Thereupon, pseudo-colour images were generated using the resulting lipid, protein, glycogen, and nucleic acids unmixing coefficients and plotted across the scan area to create 2D biochemical maps. Finally, the z-stack of biomolecular distribution maps were loaded into ICY 2.0.3.0 for rendering of 3D quantitative chemical images.

#### Quantitative volumetric Raman imaging (qVRI) for neratinib assessment in 3D PHH and 3D iHLC

Due to neratinib’s intrinsic fluorescence under 532 nm excitation, we employed a 785 nm laser as alternative Raman excitation source. The limited spectral range of 785 nm near-infrared detector (400 – 2300 cm^-1^) as compared to 532 nm Raman system (0 – 3600 cm^-1^), prevented spectral normalization to water signal (3,400 cm^-1^). A rolling circle baseline correction (shape size: 300) algorithm was employed to remove any other non-specific background signal artefacts (i.e., intrinsic cell fluorescence). Standard normal variate scaling was utilized to account for sample-to-sample and depth-dependent signal variations. Endmembers were extracted as references, and all were normalized accordingly before linear combination unmixing as previously described. The main difference here is the deconvolved coefficients no longer contain quantitative concentration information as previously described for qRamanomics. Regardless, the relative abundance (now in arbitrary units, a.u.) of each biomolecular analyte may be mapped throughout the specimens. Ilastik^41^ machine learning software was used to train a random forest model to classify subcellular regions of interest as cytoplasm, lipid droplets, drug/metabolite deposits, and nuclei and segment the 3D images accordingly. It should be noted that although this method offers volumetric insight into the size and abundance of intracellular features, it lacks the ability to quantify absolute local concentrations throughout the specimen. Finally, 3D object analysis was performed using ImageJ to generate particle size distribution measurements.

#### Drug treatment of 3D PHH/3D iHLC

Neratinib (catalogue no. S2150), nilotinib (catalogue no. S1033), ketoconazole (catalogue no. S1353), amiodarone HCl (catalogue no. S1979) and fluticasone propionate (catalogue no. S1992) were obtained from Selleckchem, Methadone stock solution (1 mg/ml) was provided by Department of Chemistry, University of Oslo. Stock solutions of drugs were prepared in DMSO in concentration 10 mM. 3D PHH (at day 7 after thawing) and 3D iHLC (after 24 days of differentiation) were incubated with indicated compounds for 24 h and 48 h, diluted in the concentration 10 μM in serum-free William's E media, supplemented with 1 % (v/v) ITS, 0.1 μM Dexamethasone. Control organoids were incubated in the same medium with 0.1 % (v/v) DMSO.

#### RNA extraction and real-time PCR (PCR)

RNA was isolated using RNeasy Micro kit (Qiagen) or TRIzol reagent (Thermo Fisher) according to the manufacturer’s protocol. RNA concentration and purity was determined using NanoDrop ND-1000 spectrophotometer (Thermo Fisher Scientific). cDNA was synthesized using High-Capacity cDNA Reverse Transcription Kit (Thermo Fisher Scientific, catalogue no. 4368814). Gene expression analysis was performed using a TaqMan Universal mix on a TaqMan ViiA7 Real Time PCR System. Used primers are listed in the Table S1. PPIA and GAPDH were used as endogenous control. Level of expression of genes of interest were quantified by ddCt with normalization to iHLC differentiated from the WTSIi028-A iPSC line (iHLC_3), and with normalization to control organoids for drug treatment. Data represent three donor PHH samples, and iHLC differentiated from 3 cell lines.

#### ELISA

Albumin content in the supernatant media was assayed with Human Albumin ELISA Quantitation Set (Bethyl Laboratories, catalogue no. E88-129). For the comparison between drug treated groups with control PHH or iHLC albumin concentration was normalized to the 3D spheroid/organoid total area as determined from bright field imaging using Fiji software. For the comparison between iHLC and PHH albumin concentration was normalized to the total protein content, Pierce™ BCA Protein Assay Kit (Thermo Fisher Scientific) according to the vendor instruction.

#### Immunofluorescence staining and microscopy

Organoids were fixed in 4% (w/v) PFA for 30 min on the orbital shaker. Each step was followed by 3 washings (each 5 min) in DPBS using an orbital shacking. Permeabilization and blocking was performed by incubation in PBS with 1% (w/v) BSA (Sigma Aldrich), 0.2% (v/v) Tritox-X100 (Sigma Aldrich) and 0.5% (v/v) DMSO at RT for 2 h on the orbital shaker. Staining with primary antibodies was performed for 24 h (at 4°C) with subsequently 2 h incubation with secondary antibodies (Jackson ImmunoResearch, West Grove, PA) diluted with 1 % (w/v) BSA, 0.1 % (v/v) Tritox-X100 in PBS. Antibodies (Ab) used in this study are provided in the key resource table. Nuclear counterstaining was performed with 1 μg/mL Hoechst 33258 (Sigma Aldrich) for 5 min at RT. Confocal microscopy was performed on a Zeiss 700 laser scanning confocal microscope and Andor Dragonfly Spinning Disk Confocal microscope using standard filters sets and laser lines with a 40x and 63x oil immersion objective. For the detection of neratinib fluorescence, we used imaging with excitation by UV at 350 nm and emission in far-red spectrum, 650-670 nm that allowed distinguishing between neratinib and Hoechst 33258 signals. Images were acquired using Zen software (Zeiss) as Z-stacks with 2 μm spacing between stacks and Dragonfly software with 0.5 μm spacing correspondingly. The confocal images were analyzed using Fiji software.^14^ Confocal images are displayed as a resulting Z-stack of half of the spheroid.

#### Viability and hepatotoxicity

ATP content was evaluated using Cell Titer-Glo® 3D Cell Viability Assay (Promega) according to manufacturer’s instructions. Briefly, 50 μl of the reagent was added to individual spheroids in 100 μl of culture medium. To facilitated lysis, organoids were vortexed for 1 min and the plate was incubated at 37°C in 5% CO_2_ for 20 min with subsequent luminescent signal measurement using GloMax® Multiplus Plate Reader/Luminometer (Promega). The changes in viability are represented as % compared to viability of control spheroids/organoids. The viability of organoids after 48 h of incubation with tested compounds was visualized using a LIVE/DEAD® assay (Thermo Fisher Scientific) as described by the manufacturer. Briefly, organoids were washed in DPBS and incubated for 30 min at 37°C in a 5 % CO_2_ incubator in 1 mL of culture media containing 1 μL of calcein AM solution and 5 μL of ethidium homodimer-1 solution. Stained spheroids/organoids were analyzed using a fluorescence microscope (Zeiss).

#### Cytochrome CYP3A4 and CYP1A2 activity

Cytochrome CYP3A4 and CYP1A2 enzymatic activities of 3D PHH and 3D iHLC were measured using P450-Glo™ Assay with Luciferin-IPA (catalogue no. V9001) and Luciferin-1A2 (catalogue no. V8771) correspondingly (Promega, Sweden). For the induction of CYP3A4 activity, organoids were treated with 50 μM of rifampicin for 24 h prior the analysis. For the induction of CYP1A2 activity organoids were treated with 100 μM of omeprazole for 24 h prior the analysis. Relative luminescence was normalized to total protein content, measured by Pierce™ BCA Protein Assay Kit (Thermo Fisher Scientific) according to the vendor instruction.

#### Transporter activity

Spheroids/organoids were incubated with 10 μM 5(6)-carboxy-2′,7′-dichlorofluorescein diacetate (CDFDA) (Sigma Aldrich, catalogue no. 21882) for 30 min at 37°C in the 5 % CO_2_ atmosphere. Nuclei were counterstained with 1 μg/mL Hoechst 33342. Cultures were washed with PBS containing calcium and magnesium. Imaging was performed in William's E media without phenol red, but containing 5 mM of HEPES using a Zeiss LSM 700 confocal microscope after 5, 15 and 30 min after washing at 37°C.

#### Phospholipidosis assay and neutral lipids staining

The phospholipids accumulation was monitored in real time using HCS LipidTOX™ Phospholipidosis Detection Reagents (PLD) (Thermo Fisher Scientific, catalogue no. H34350) and detection by Incucyte live imaging visualization system. For real-time experiments spheroids were generated in 96 wells ultra-low attachment U-bottom plate with initial plating density of 1000 cells per well. PLD accumulation data is represented by mean fluorescence signal per well. Total accumulation of PLD after 48 h of drug exposure was verified using Andor Dragonfly Spinning Disk Confocal microscope. Z-stack of half of spheroid was taken with spacing 1 μm. Neutral lipids were stained using HCS LipidTOX™Red Neutral Lipids (excitation/emission maxima ∼577/609 nm, Thermo Fisher Scientific, catalogue no. H34476) and visualized using Andor Dragonfly Spinning Disk Confocal microscope.

#### Proteomic liquid chromatography-tandem mass spectrometry (LC-MS/MS) analysis

Pelleted iHLC organoids generated from 3 cell lines (iHLC_1: WTC-11, WiCell; iHLC_2: WTSIi013-A and iHLC_3: WTSIi028-A, Wellcome Trust Sanger Institute) and cryopreserved primary human hepatocytes (Gibco, lot HU8287 (PHH_3)) (all ∼100 000-300 000 cells) were washed once with DPBS (final volume ∼15 μL), before lysing and digestion. Sample preparation was performed according to the protocol for sample preparation by Easy Extraction and Digestion (SPEED) by Doellinger et al.,^15^ with a modified reduction and alkylation step: reduction was performed by adding DL-dithiothreitol (Merck, catalogue nr. D5545) to a final concentration of 10 mM before incubation at 56°C / 900 rpm for 25 min in a thermoshaker (Grant instruments), and alkylation was performed by adding iodoacetamide (Merck, catalogue no. I1149) to a total concentration of 20 mM before incubation at room temperature / 900 rpm for 30 min in the thermoshaker (in the dark). Samples were digested with 6 μg trypsin () overnight in the thermoshaker at 37°C / 700 rpm. To terminate protease activity, trifluoroacetic acid was added to a final concentration of 1 % (v/v) and the peptide extracts were concentrated to dryness using a Concentrator plus from Eppendorf (Hamburg). The dried extracts were dissolved in 100 μL LC-MS grade water with 0.25 % (v/v) heptafluorobutyric acid before sample clean up. Cleanup was performed using 100 μL BondElute C18 solid-phase extraction columns from Agilent (Santa Clara) according to the attached protocol and eluted into 100 μL acetonitrile/water/formic acid (60/40/0.1, v/v/v) in 1.5 mL Eppendorf Protein-LoBind tubes. The two aliquots of each sample were combined and concentrated to dryness in the Concentrator plus, and the final peptide extracts were dissolved in 4 μL LC-MS grade water containing 0.1 % (v/v) formic acid.

The protein extracts were analyzed by LC-MS/MS using the timsTOF Pro (Bruker Daltonik) mass spectrometer which was coupled online to a nanoElute nanoflow liquid chromatography system (Bruker Daltonik) via a Captive Spray nanoelectrospray ion source. The peptides were separated on a reversed-phase C18 column (25 cm x 75 μm, 1.6 μm, Ion Optics (Fitzroy) at 50°C). Mobile phase A contained LC-MS grade water with 0.1 % (v/v) formic acid, and acetonitrile with 0.1 % (v/v) formic acid was used as mobile phase B. The peptides were separated by a linear gradient from 0 – 35 % mobile phase B over 54 min at a flow rate of 300 nL/min at a column temperature of 50°C. MS acquisition was performed in data-dependent acquisition parallel accumulation-serial fragmentation (DDA-PASEF) mode. An injection volume of 2 μL was used.

The LC-MS data were searched against the human UniProt database (20,431 entries), with PEAKS X+ software version 10.5 (Bioinformatics Solutions). The following parameters were used: digestion enzyme, trypsin; maximum missed cleavage, 1; fragment ion mass error tolerance, 0.03 Da; parent ion error tolerance, 15 ppm. Oxidation of methionine, carbamidomethyl formation on cysteines, and acetylation of the N-terminus was specified as variable modifications and the maximum number of posttranslational modifications (PTMs) was set to 2. A false-discovery rate of 1 % was applied to the datasets. Label-free quantification (LFQ) using the PEAKS X+ software was performed using proteins containing at least 1 peptide in both groups (iHLC_1 and PHH) with a significance ≥0 and FDR of 5 %. Peptides were filtered, retaining peptides with a 2≤ charge≤5, quality ≥0, and area ≥0. Normalization to the total protein intensity was performed (intensity of the PHH reduced by a factor of 5.6 compared to that of iHLC_1).

#### Drug metabolism in microsomes

Human liver microsomes (XTremme Pool 200 Human, Tebu-bio, Lot nr: 1710084) were stored at -80°C. NADPH regeneration solution (final concentration 1.3 mM NADP+, 3.3 mM glucose-6-phosphate, 0.4 U/mL glucose-6-phosphate dehydrogenase, 3.3 mM MgCl_2_) and human liver microsomes (final concentration 1 mg protein/mL) were pre-incubated to 37°C for 15 min in a shaking water bath. The reaction was initiated by addition of neratinib or amiodarone (final concentration of 5 μM in a total volume of 100 μL) and stopped after 0, 20, and 60 min by addition of ice-cold formic acid (FA, final concentration 0.11 M). The samples were centrifuged at 14500 × g and 4°C for 10 min and the supernatants were transferred to autosampler vials. Drug degradation control samples, without human liver microsomes, were performed in parallel to evaluate the stability of neratinib at the incubation conditions.

#### Neratinib and amiodarone metabolites detection by LC-MS

PHH spheroids were incubated in 5 μM neratinib or 10 μM amiodarone in serum-free William’s E medium supplemented with 0.1 μM dexamethasone and 1% (v/v) ITS for 6 and 24 h. Metabolism was stopped by adding FA to a final concentration of 0.11 M, and the plates were frozen at −80°C. In parallel, samples of cell medium without organoids (n = 3) were used as drug degradation control samples. The samples were centrifuged at 14500 × g and 4°C for 10 min. The supernatant was diluted 10x in 0.1% (v/v) FA prior to analysis. The pellet was washed three times in PBS, followed by the addition of 100 μL type I. Rapidly, pellet and liquid were transferred to Eppendorf tubes and then placed in an ultrasonic bath for further preparation, applying a variant of procedure described before,^16^ to reduce sample heating; in the bath, the cells were subjected to ultrasonic treatment for 30 s on/30 s off 10 times, taking a total of 10 min. Detection of neratinib metabolites was achieved with an Agilent 1100 series pump equipped with an Agilent 1200 autosampler from Agilent Technologies. The autosampler and pump was coupled to Quantiva (triple quadrupole) MS with an electrospray ionization (ESI) interface from Thermo Fisher Scientific. Separation was performed using a HotSep® Sunniest C18 analytical column (150 x 0.5 mm, 3 μm particles and 120 Å pores) from GT Septech Teknolab (Ski, Norway). The Agilent 1100 series pump was equipped with two solvent compartments (A and B), where A contained 5 mM ammonium format pH 3.1 (w/v) and B contained 0.1 % FA in LC-MS grade water and acetonitrile (5/95, v/v). A linear gradient was applied ranging from 20 % to 80 % B in 6 min at a flow rate of 15 μL/min, and the injection volume was 3 μL. The ion spray voltage was set to 3.5 kV, the sheath- and aux gas was set to 7 arb and 5 arb, and the vaporizer temperature was set to 33°C. The MS operated in positive mode, with multiple reaction monitoring (MRM) transitions of neratinib and metabolites obtained from neratinib metabolite detection in neratinib incubated human liver microsomes. The neratinib metabolite identification quality control was based on the characteristic Cl isotope intensity ratio of 3:1, and the retention time order and MS/MS fragments were matched with the study of Liu et al.^54^ The MRM transitions and collision energies (ce) for neratinib, Peak 3 (*m/z* 557.2> 112.2, 512.2, and 521.3 at 20 V), Peak 1 (*m/z* 466.2> 112.8, 393.1, and 421.2 at 20 V), Peak 2 (*m/z* 543.2> 353.0 at 37 V, and 543.2> 446.1, and 507.5 at 20 V), Peak 4 (*m/z* 573.2> 464.3, 528.3, and 111.9 at 20 V). Solvent gradients and MS acquisition was controlled by the Agilent LC software (Chemstation).

Amiodarone metabolite detection was achieved with ESI-MS (triple quadrupole Quantiva, Thermo Fisher Scientific), and direct injection using a Fusion 101 syringe pump from ChemyxInc (Stafford, TX). Here, the flow rate was 5 μL/min, the ion spray voltage was set to 4.5 kV, the sheath- and aux gas was set to 3 arb and 5 arb, and the vaporizer temperature was set to 33°C. The MS operated in positive mode.

### Quantification and statistical analysis

#### Raman spectral preprocessing

All acquired Raman spectra were preprocessed in WITec ProjectFIVE 5.2 and Matlab following the same pipeline on a per pixel basis: cosmic ray removal (filter size: 4; dynamic factor: 4.1), setting minimum value in Rayleigh region (-150 - 50 cm^-1^) to zero (detector dark current to zero), normalization setting the main water peak average value (3220−3420 cm^−1^) equal to one, matrix/medium background subtraction using a matrix blank (i.e., 1 % agarose (w/v) in PBS), and rolling circle baseline correction (shape size: 300) to remove any other non-specific background signal artifacts. Finally, all spectra were cropped from 400 - 3100 cm^−1^while also ignoring the biological "silent region" from subsequent unmixing (1800 - 2700 cm^−1^).

#### Spectral unmixing via linear combination modeling

The spectroscopic unmixing problem for any Raman spectrum can be described as

(Equation 1) $$y_{i}=X\beta_{i}+e_{i}$$

or in matrix form as

(Equation 2) Y=XB+E

Where $y_{i}$ is the *i−th m × 1* sample spectrum of the unfolded Raman image dataset; X is the *m × n* calibration dataset matrix; $\beta_{i}$ the *i−th n × 1* mixing coefficient vector and $e_{i}$ the *i−th m × 1* unmodeled residual vector. Within this linear algebra framework, to obtain $\beta_{i}$ estimations,

(Equation 3) $$\hat{y_{i}}=X\hat{\beta_{i}}$$

as

(Equation 4) $$e_{i}=y_{i}-\hat{y_{i}}$$

Thus

(Equation 5) $$\hat{\beta_{i}}={\left(X^{T}\;X\right)}^{-1}\;X^{T}\;y_{i}$$

where $\hat{\beta_{i}}$ and $\hat{y_{i}}$ are the algorithmic model estimations of $\beta_{i}$ and $y_{i}$ respectively. There are several ways of computing Equation 5, although for simplicity and interpretability, in this study we use ordinary least squares with non-negativity constraint on $\hat{\beta_{i}}$. Once the Raman spectral unmixing is performed, the estimated coefficient values ($\hat{\beta_{i}}$) provide a map/image of the biochemical concentration distribution of each endmember throughout the sample.

Using this mathematical formulation as a mean to estimate chemical concentrations from Raman spectra carries an implicit assumption: the relationship between the concentration of an analyte and its corresponding Raman signal must be linear. Consequently, mixtures spectra are considered as ideal additions of each component’s Raman fingerprint scaled by a coefficient that is directly correlated with its concentration. This assumption has been proved in several different scenarios,^5^^,^^6^ however, when dealing with biochemical samples, small alterations to the individual concentration-Raman signal patterns may arise due to molecular interactions among bio-molecular species. To account for these potential effects within our linear model, chemical mixtures were also included in the calibration set at different concentration combinations.

#### Statistics

Statistical analyses and graphs generation were performed using GraphPad PRISM 7 (GraphPad Software Inc.). Unless specifically stated, a two-tailed, paired *t*-test (with unequal variances) was applied for the comparison of two groups. For more than two groups, a one-way ANOVA analysis was applied. The data are presented as mean ± SD. Statistical significance was assigned as not significant (NS) p > 0.05; ∗p ≤ 0.05; ∗∗p ≤ 0.01; ∗∗∗p ≤ 0.001; ∗∗∗∗p ≤ 0.0001.

## Data Availability

• Raw data and scripts are available upon request from rdm-enquiries@imperial.ac.uk. • All original code has been deposited at GitHub and is publicly available via Zenodo: https://doi.org/10.5281/zenodo.7628912. The DOI is also listed in the key resources table. • Any additional information required to reanalyze the data reported in this paper is available from the lead author (Prof. Molly M. Stevens (m.stevens@imperial.ac.uk)) upon request.

## Supplemental information

Document S1. Figures S1–S7 and Table S1

Document S2. Article plus supplemental information
