# Supplementary material for: Quantitative chemometric phenotyping of three-dimensional liver organoids by Raman spectral imaging
Source: Cell Rep Methods. 2023 Mar 31;3(4):100440. doi: 10.1016/j.crmeth.2023.100440 (PMC10162950; doi:10.1016/j.crmeth.2023.100440)
Supplement: Document S1. Figures S1–S7 and Table S1 [file mmc1.pdf]

Cell Reports Methods, Volume *3*

Supplemental information

Quantitative chemometric phenotyping of three-dimensional liver organoids by Raman spectral imaging

Vernon LaLone, Aleksandra Aizenshtadt, John Goertz, Frøydis Sved Skottvoll, Marco Barbero Mota, Junji You, Xiaoyu Zhao, Henriette Engen Berg, Justyna Stokowiec, Minzhi Yu, Anna Schwendeman, Hanne Scholz, Steven Ray Wilson, Stefan Krauss, and Molly M. Stevens

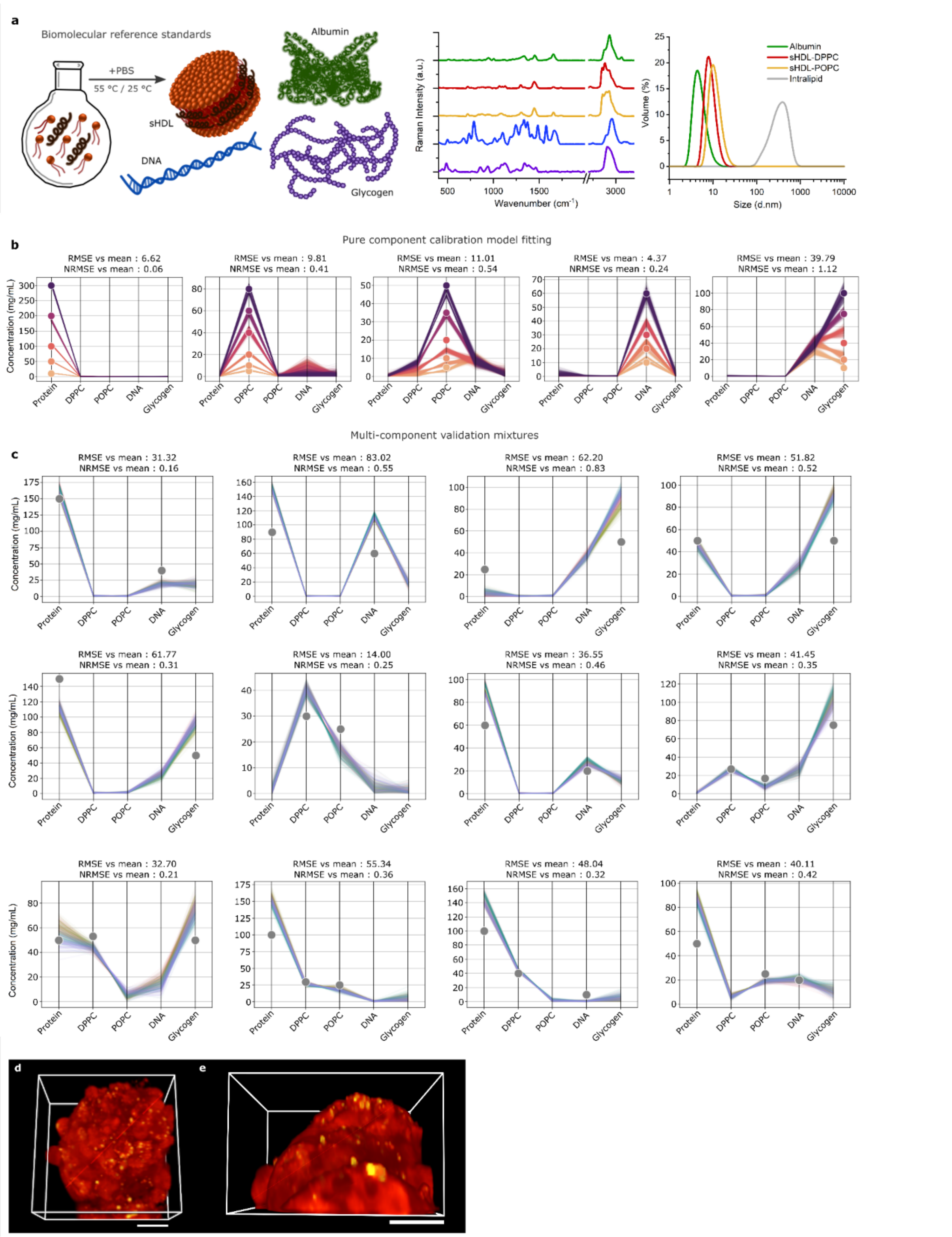

***Figure S1. Method validation for quantitative compositional analysis in three-dimensional biospecimens using Bayesian approach to characterise model confidence, related to Figure 1. a**, sHDLs enable mixing of lipid species with other classes of biomolecules in aqueous mediums. Reference spectra for each major component normalized to maximum peak height. Particle size distribution of sHDLs compared with albumin solutions and intralipid emulsions by DLS. **b,** Results for pure component calibration model fitting. Datapoints reveal ground truth concentrations of analytes and shaded lines show confidence boundaries of various measurement sets. **c**, Multi-component mixtures provide measure of model confidence and accuracy to quantitatively deconvolute biomolecule spectral signatures from physiologically-relevant concentration realms. **d, e,** 3D image generated from preprocessed spectra showing total signal acquired in high wavenumber region (spectra integrated from 2800–3000 cm^{-1}) for the whole PHH spheroid (d, Scale bar = 50 µm) and its segment – at the subcellular resolution (e, Scale bar = 10 µm).*

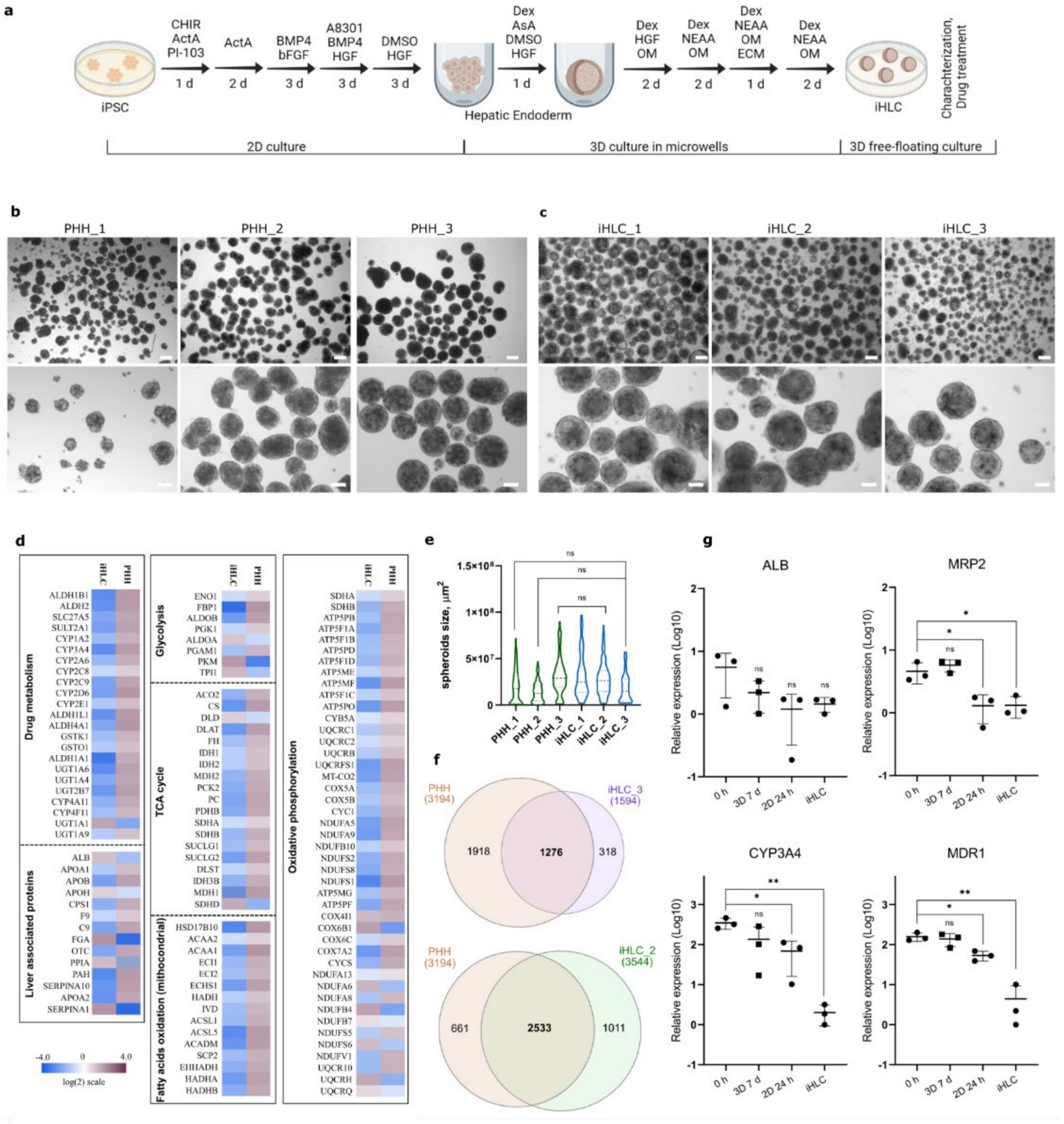

Figure S2. Comparison of iHLC and PHH, related to Figure 1 and Figure 2. a, *Schematic representation of differentiation protocol.* ***b-c****, Representative bright field images of PHH spheroids from 3 donors, and iHLC generated from 3 hiPSC lines. Scale bars 100 µm.* ***d****, Heat map illustrating relative abundance of proteins in PHH_3 and iHLC_1. Proteins were grouped into groups related to drug-metabolism, Glycolysis, TCA cycle, oxidative phosphorylation and fatty acids phosphorylation, as well as general liver-specific proteins.* ***e****, Size distribution of PHH spheroids and iHLC organoids, measured as an area of 3D models sections from bright field images (n>90 for each group, one-way ANOVA).* ***f****, Venn diagram of proteins detected in PHH_3 and iHLC_2 and iHLC_3 organoids.* ***g****, Relative expression of selected hepatic markers in iHLC organoids, freshly thawed (0 h) PHH, PHH after 7 days and 24 h of 3D and 2D culture correspondingly. n = 3 donors for PHHs, n = 3 cell lines for iHLC organoids. Significance was compared with PHH after 24 h in 2D culture using one-way ANOVA, *p<0.05, **p<0.05.*

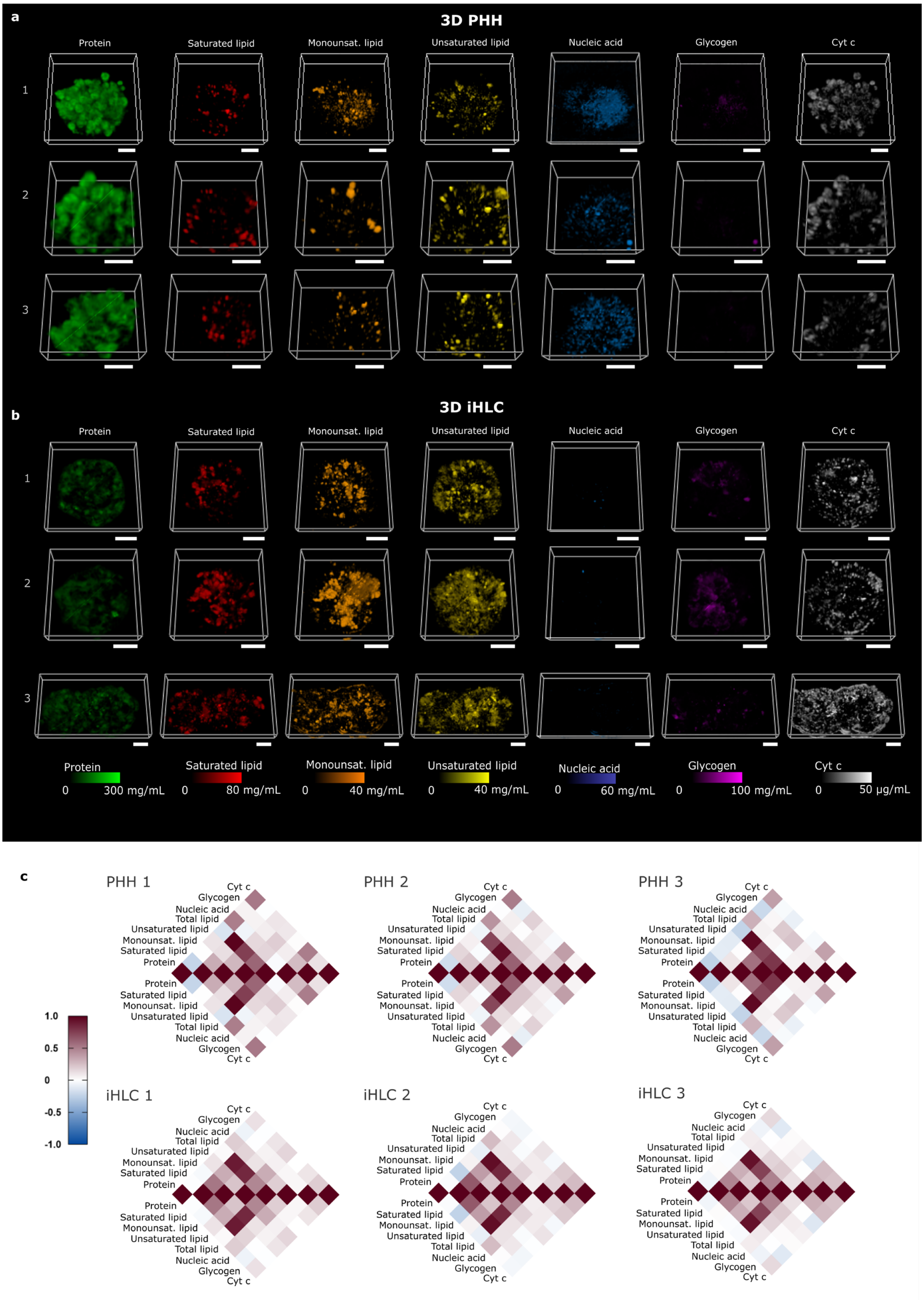

Figure S3**. qRamanomics reveals repeatable compositional phenotypic changes between different spheroids in same treatment group, related to Figure 2. a-b*, *High-content quantitative Ramanomic imaging reveals distribution of molecular content throughout 3D PHH spheroids (a) from a single donor (n = 3) and 3D iHLC organoids (b) (n = 3). Scale bars = 50 μm.* ***c*, Pearson's correlation chemometric heatmaps illustrate colocalization of various molecular components and % difference between PHH and iHLC.*

a

Drug	Drug Class	Hepatotoxicity likelihood score[38]	Mechanism of hepatotoxicity
**Amiodarone**	Group III antiarrhythmic	A (well-established cause of clinically apparent liver injury)	Induces microvesicular steatosis phospholipidolisis[39, 52, 53]
**Nilotinib**	BCR-ABL tyrosine kinase inhibitor	D (possible rare cause of clinically apparent liver injury)	NA
**Fluticasone-propionate**	Inhaled corticosteroid	No liver injury effect	NA
**Ketoconazole**	Azole antifungal	A (well-established cause of clinically apparent liver injury)	Inhibitor of CYP3A4[32]. Hepatotoxicity may correlate with inhibition of mammalian sterol synthesis[33,34]
**Methadone**	Narcotic analgesic	No liver injury effect	NA
**Neratinib**	EGFR & HER2 inhibitor	E* (unproven but suspected cause of clinically apparent injury)	NA

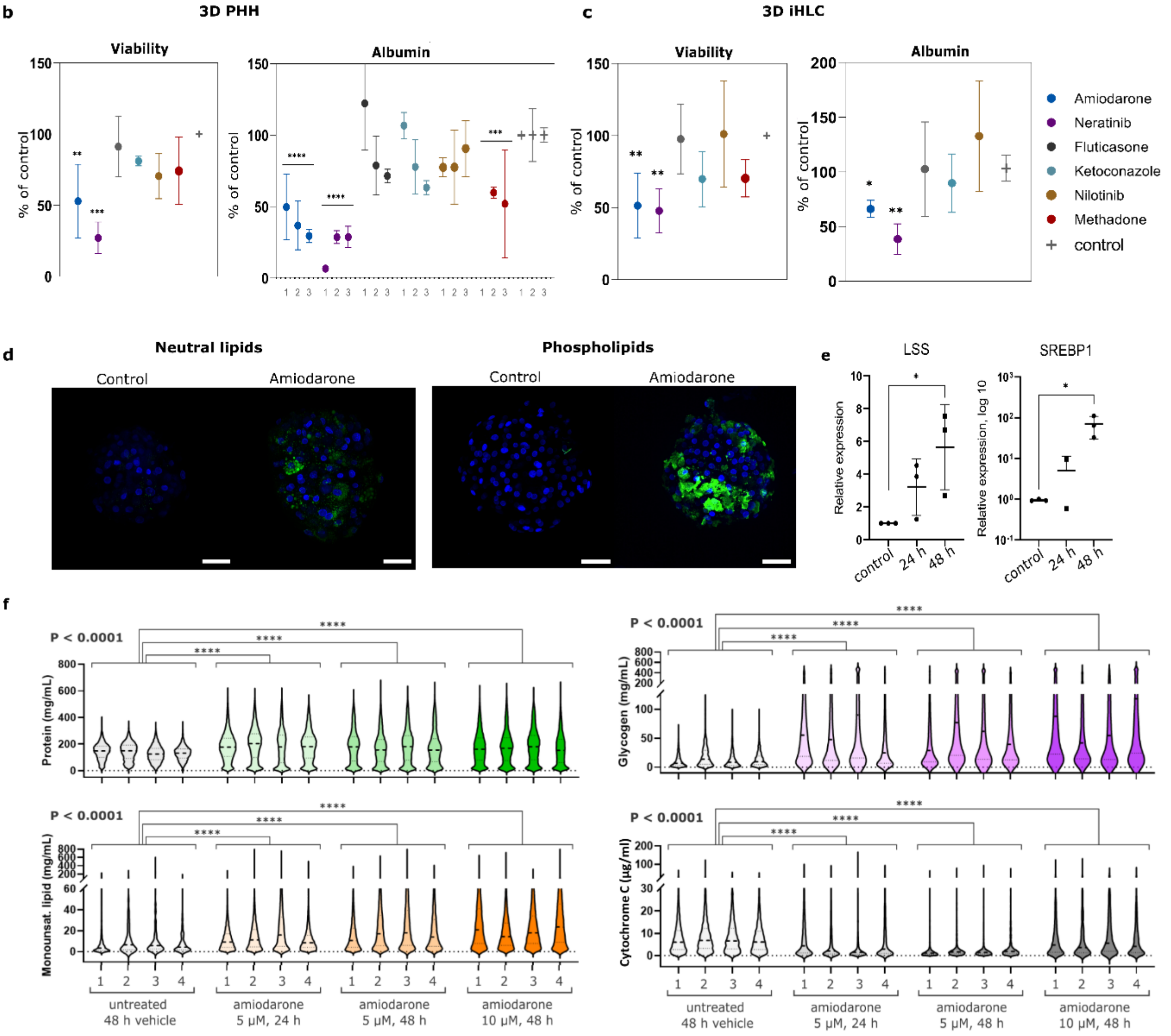

Figure S4. Changes in 3D liver representations in response to drug exposure, related to Figure 3. a, *Table of tested compounds.* ***b-c,*** *Drug-induced changes in the viability (measured as ATP content) and albumin secretion level of PHH spheroids (b) and iHLC organoids (c) after 48 h incubation with selected compounds. Data represented in % of changes from control as mean±SD, n = PHH from 3 donors, n = iHLC organoids from 3 independent differentiation experiments. *p<0,05, **p<0,01.* ***d,*** *Representative confocal images of FITC-labelled phospholipids and neutral lipids in PHH spheroids incubated for 48 h with amiodarone (10 µM) and vehicle (control). Scale bars = 50 µm.* ***e,*** *Relative expression of lanosterol synthase (LSS), sterol regulatory element-binding transcription factor 1 (SREBP1) in PHH spheroids after 24 and 48 h of incubation with amiodarone (10 µM). n = 3 donors for PHHs, significance was compared with untreated PHH, using one-way ANOVA, *p<0.05.* ***f,*** *Analysis demonstrates repeatability across multiple spheroids from each sample group. Nested one-way ANOVA followed by Kruskal-Wallis multiple comparisons were significant (n = 137,374 protein, 118,193 monounsaturated lipid, 124,697 glycogen, and 60,003 cyt c measurements from 16 different 3D PHH spheroids).*

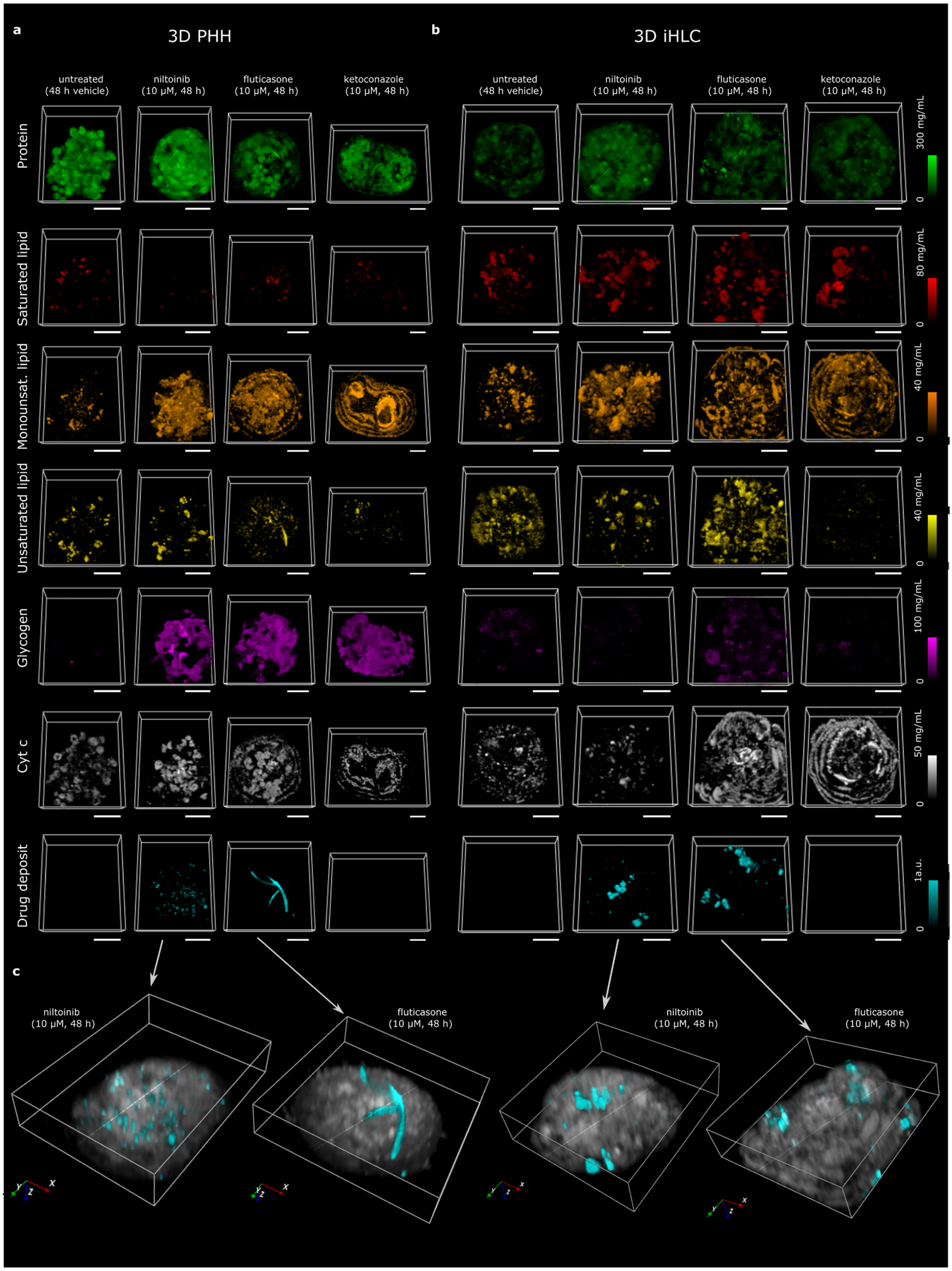

Figure S5. qRamanomics reveals chemotypic changes in response to drug treatment for PHH spheroids and iHLC, related to Figure 3 and Figure 4. a, b, *High-content quantitative Ramanomic imaging reveals distribution of molecular content throughout 3D PHH spheroids from a single donor (n = 3) (a) and iHLC organoids from 1 cell line (n = 3) (b). Scale bars = 50 µm.* ***c,*** *3D renderings of drug and/or drug metabolite deposits (shown in cyan) detected in PHH spheroids (left) and iHLC organoids. The biomolecular matrix (i.e., protein, total lipid, and glycogen) was combined and is shown in grey (arbitrary intensity units) to reveal clear distinction between endogenous biomolecules and xenobiotic compounds present in specimens. Scale bars = 50 µm.*

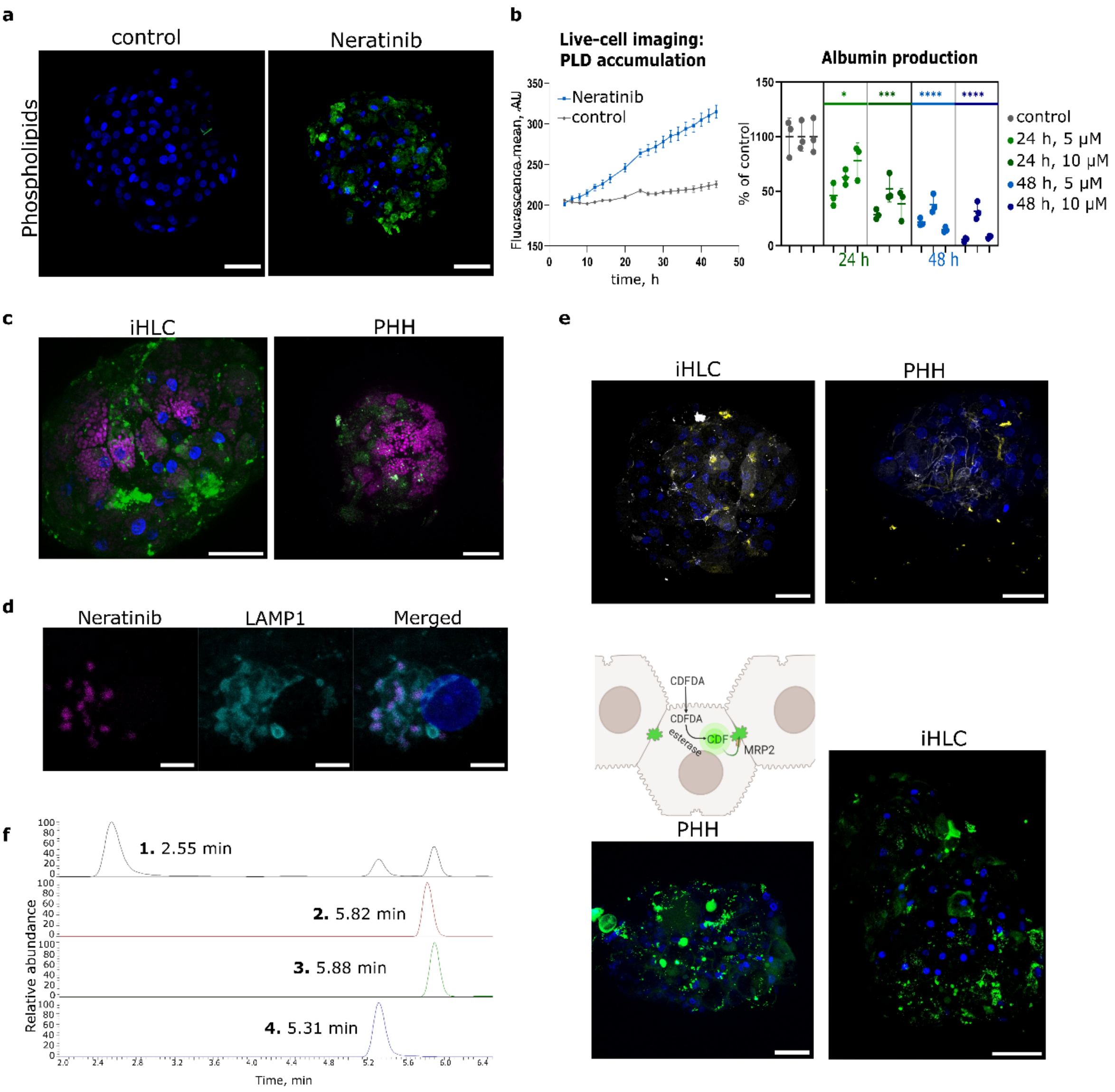

Figure S6. Lysosomal accumulation of neratinib and its metabolites is associated with phospholipidosis and hepatotoxicity, related to Figure 4. a, *Intracellular accumulation of phospholipids in PHH spheroids (confocal image after 48 h) and iHLC organoids (live cell imaging, Incucyte platform) in response to the incubation with 5 µM Neratinib. Scale bars = 50 µm.* ***b,*** *Time-and concentration-dependent Neratinib-induced decrease of albumin production in PHH spheroids from 3 donors. n = 3 replicates for each donor. Comparison performed using nested one-way ANOVA.* ***c,*** *Fluorescence of Neratinib and/or associated metabolites in primary hepatocytes (PHH) and iHLC in 3D organoids. Scale bars = 50 µm.* ***d,*** *Accumulation of Neratinib and/or associated metabolites in LAMP+ lysosomes. Scale bars = 5 µm.* ***e,*** *Bile canaliculi-like structures with functional transporter MRP2 in PHH spheroids and iHLC organoids visualized by immunofluorescent staining for ZO1 and MRP2, and CDFDA analysis. Scale bars = 50 µm.* ***f,*** *Representative chromatograms of Neritanib and selected metabolites. Peak 1 (M3 described reference 43): Selected reaction monitoring (SRM) = 466.2 [M-H+] à 112.8, 393.1, 421.2. Peak 2 (M10 described in reference 53): SRM = 543.200 [M-H+] à 353.0, 446.1, 507.5. Peak 3 (Neritanib): SRM = 557.2 [M-H+] à 112.2, 512.2, 521.3. Peak 4 (M12 described in reference 43): SRM = 573.2 [M-H+] à 464.3, 528.3, 111.9.*

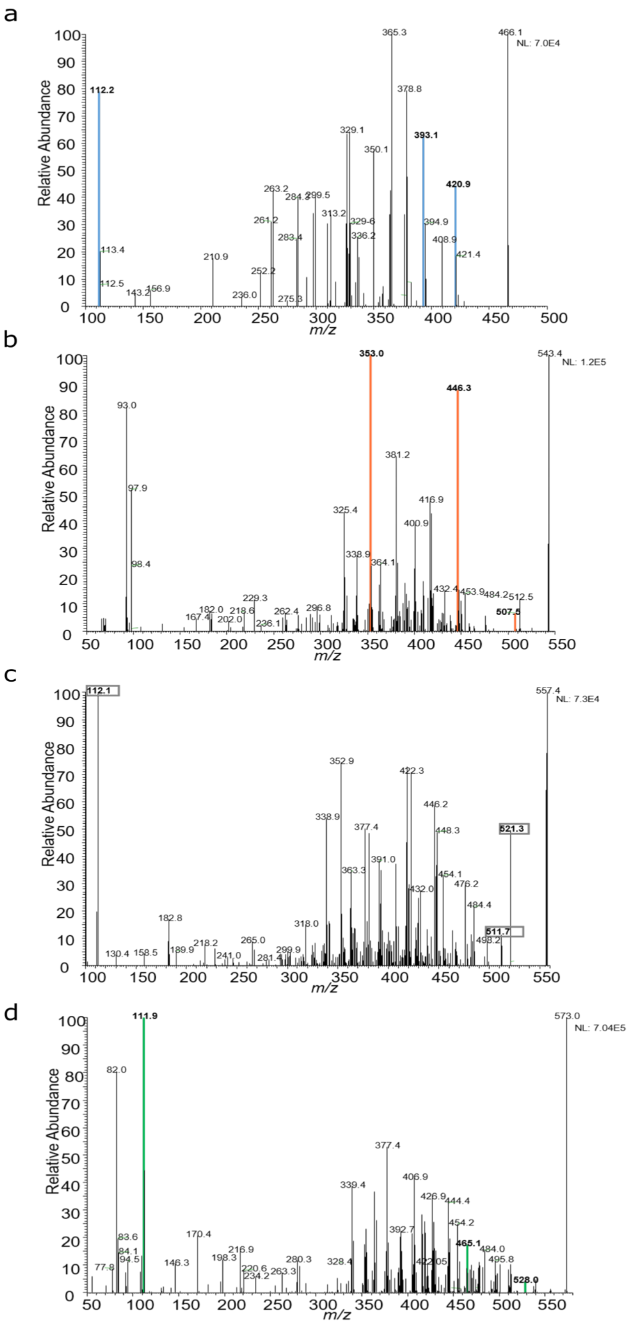

Figure S7. Representative chromatograms of neratinib and selected metabolites, related to Figure 4. a, *Peak 1 (M3 described in reference 53): Selected reaction monitoring (SRM) = 466.2 [M-H+] à 112.8, 393.1, 421.2.* ***b****, Peak 2 (M10 described in reference 53): SRM = 543.200 [M-H+] à 353.0, 446.1, 507.5.* ***c****, Peak 3 (neratinib): SRM = 557.2 [M-H+] à 112.2, 512.2, 521.3.* ***d****, Peak 4 (M12 described in reference 53): SRM = 573.2 [M-H+] à 464.3, 528.3, 111.9.*

Gene	Assay ID	Vendor
ABCB11 (BSEP)	Hs00184824_m1	ThermoFisher Scientific
ABCC2 (MRP2)	Hs00166123_m1	ThermoFisher Scientific
ABCB4 (MDR4)	Hs00240956_m1	ThermoFisher Scientific
ABCB1 (MDR1)	Hs00184500_m1	ThermoFisher Scientific
ALB	Hs00609411_m1	ThermoFisher Scientific
HNF4A	Hs00230853_m1	ThermoFisher Scientific
CYP3A4	Hs00604506_m1	ThermoFisher Scientific
CYP2e1	Hs00559367_m1	ThermoFisher Scientific
CYP1A2	Hs00167927_m1	ThermoFisher Scientific
CYP7A1	Hs00167982_m1	ThermoFisher Scientific
CYP2B6	Hs04183483_g1	ThermoFisher Scientific
CYP2C9	Hs02383631_s1	ThermoFisher Scientific
UGT1A1	Hs02511055_s1	ThermoFisher Scientific
UGT2B7	Hs00426592_m1	ThermoFisher Scientific
A1AT (SERPINA)	Hs01097800_m1	ThermoFisher Scientific
Glul	Hs01018343_g1	ThermoFisher Scientific
CPS1	Hs00157048_m1	ThermoFisher Scientific
GYS2	Hs01072354_m1	ThermoFisher Scientific
SOD2	Hs00167309_m1	ThermoFisher Scientific
SREBF1	Hs02561944_s1	ThermoFisher Scientific
GBE1	Hs00609186_m1	ThermoFisher Scientific
LSS	Hs01552331_m1	ThermoFisher Scientific
CES1	Hs00275607_m1	ThermoFisher Scientific
PCK1	Hs00159918_m1	ThermoFisher Scientific
GAPDH	Hs02786624_g1	ThermoFisher Scientific
PPIA	Hs04194521_s1	ThermoFisher Scientific
TBP	Hs00427620_m1	ThermoFisher Scientific

Table S1. List of used primers, related to the STAR Methods.
